# Supplementary material for: Improved in situ characterization of protein complex dynamics at scale with thermal proximity co-aggregation
Source: Nat Commun. 2023 Nov 24;14:7697. doi: 10.1038/s41467-023-43526-2 (PMC10673876; doi:10.1038/s41467-023-43526-2)
Supplement: Supplementary file 1 — Supplementary information [file 41467_2023_43526_MOESM1_ESM.pdf]

Supplementary information

Improved *in situ* Characterization of Proteome-wide Protein Complex Dynamics with Thermal Proximity Co-Aggregation

Siyuan Sun[1,2], Zhenxiang Zheng[1,2], Jun Wang[1], Fengming Li[1], An He[1], Kunjia Lai[1], Ruijun Tian[1], Chris Soon Heng Tan[1]*

[1]Department of chemistry, Southern University of Science and Technology;

[2]These authors contributed equally:

* To whom correspondence should be addressed. E-mail: christan@sustech.edu.cn

Supplementary information includes:

1. Supplementary Figures 1-7 (page 2-7)

2. Supplementary Figures 16-22 (page 8-14)

3. Supplementary Methods (page 15)

Supplementary Figure 1-7

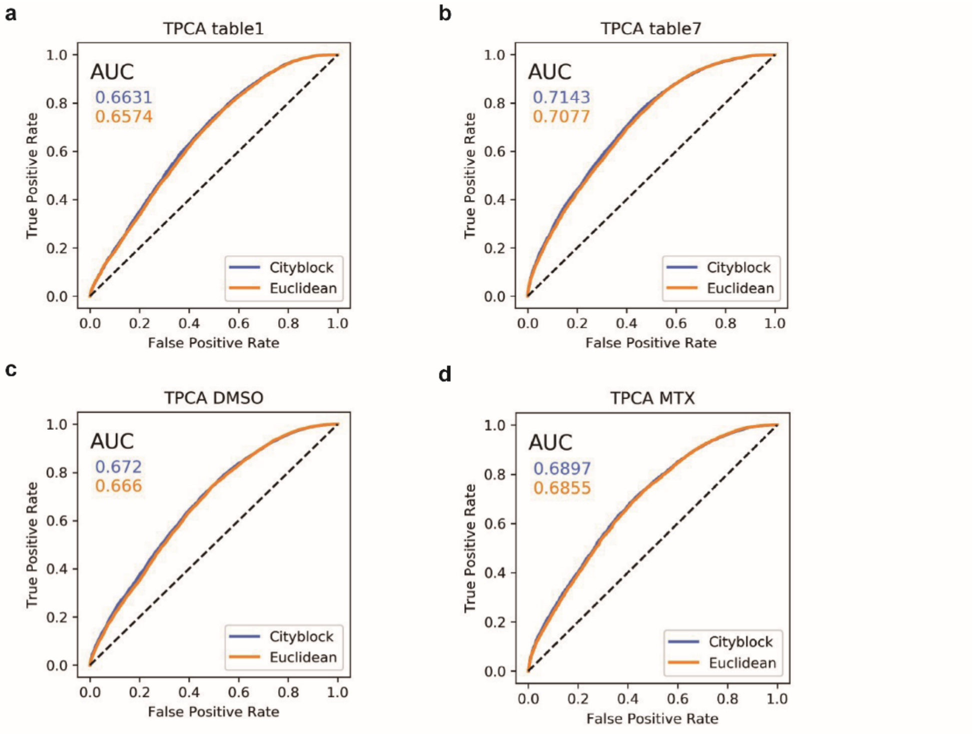

Supplementary Figure 1. AUC values are improved using Manhattan distance over Euclidean distance across different data sets. (**a-d**) The four subplots represent proteomic data in cell lysates, in intact cells, in cells treated with DMSO, and in cells treated with MTX, respectively. The proteomics data for all four conditions suggested that the Manhattan distance could improve the AUC values compared to the Euclidean distance.

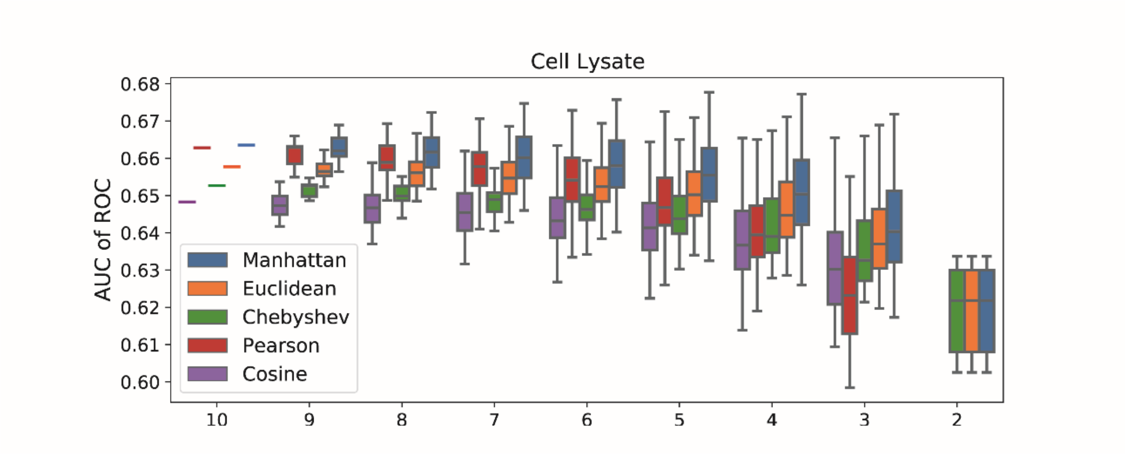

Supplementary Figure 2. Box plot of the ability of different measures in predicting PPI when used with fewer temperature points in the cell lysate data. All combinations of temperature were tested accordingly. AUC: Area Under Curve; ROC: Receiver Operating Characteristic curve. The box extends from the lower quartile to the upper quartile values of the data, with a line at the median. When n out of 10 temperature points are selected, there are $10!/(10-n)!\,n!$ unique temperature point combinations. All combinations of temperature points are tested and predictive power generally decreases with less temperature points. Pearson's distances and Cosine distances are meaningless with 2 temperature points. Data are presented as mean values +/- SEM.

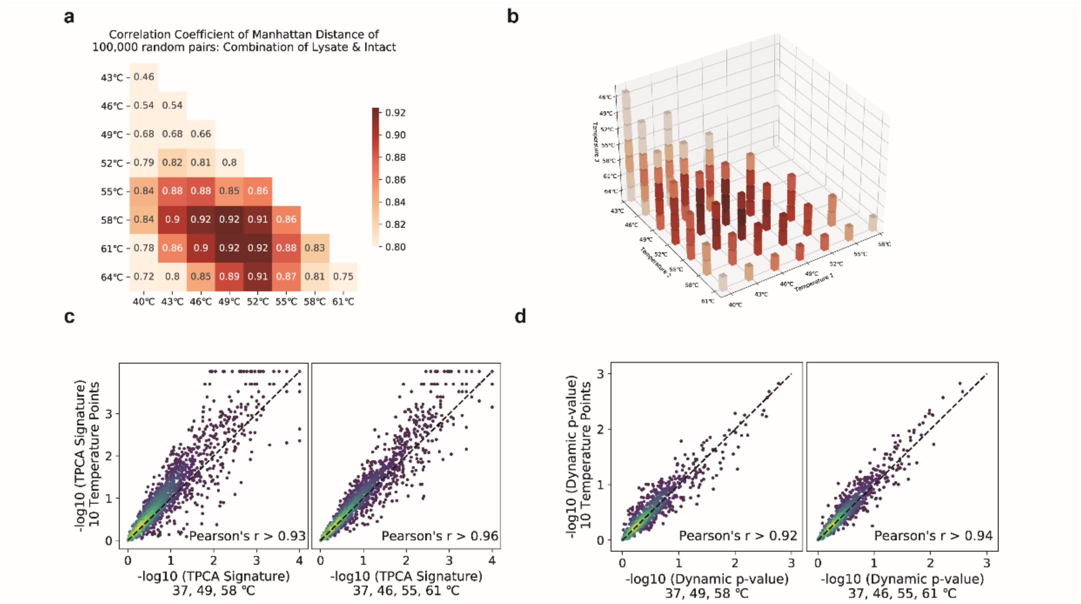

Supplementary Figure 3. Selection of optimal combinations of temperature points for TPCA profiling. (**a**) Correlation of Manhattan distances for 100,000 random protein pairs calculated using 3 temperature points and 10 temperature points. The combinations of 3 temperature points ∈ {37°C, Ta°C, Tb°C }, where Ta, Tb ∈ {40°C, 43°C, 46°C, 49°C, 52°C, 55°C, 58°C, 61°C, 64°C }. (**b**) Correlation of Manhattan distances for 100,000 random protein pairs calculated using 4 temperature points with those calculated using 10 temperature points. The combinations of 4 temperature points ∈ {37°C, Ta°C, Tb°C, Tc°C }, where Ta, Tb, Tc ∈ {40°C, 43°C, 46°C, 49°C, 52°C, 55°C, 58°C, 61°C, 64°C }. (**c**) Statistical significance of TPCA signature for CORUM complexes (p-value) quantified with 3 and 4 temperature points as compared to 10 temperature points. (**d**) Statistical significance of dynamic (modulated) TPCA signatures for CORUM complexes (TPCA Modulation Signature) quantified with 3 and 4 temperature points as compared to 10 temperature points.

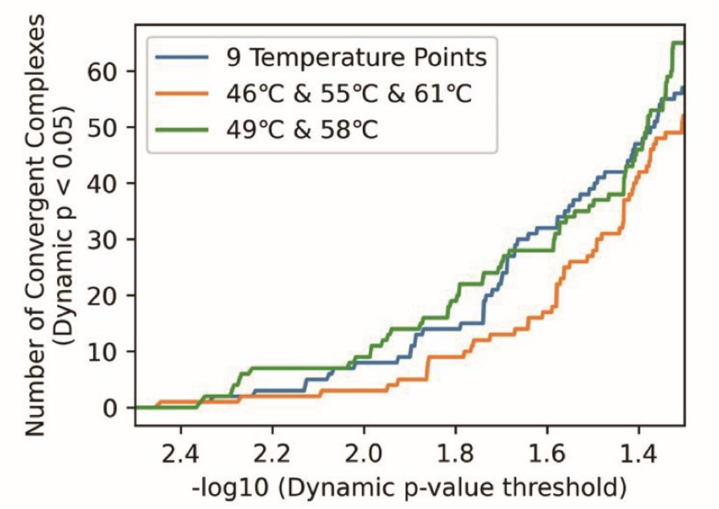

Supplementary Figure 4. Cumulative plot of the number of complexes identified with TPCA modulation signatures. Dynamic complexes identified using fewer temperature points (TPCA modulation $p < 0.05$).

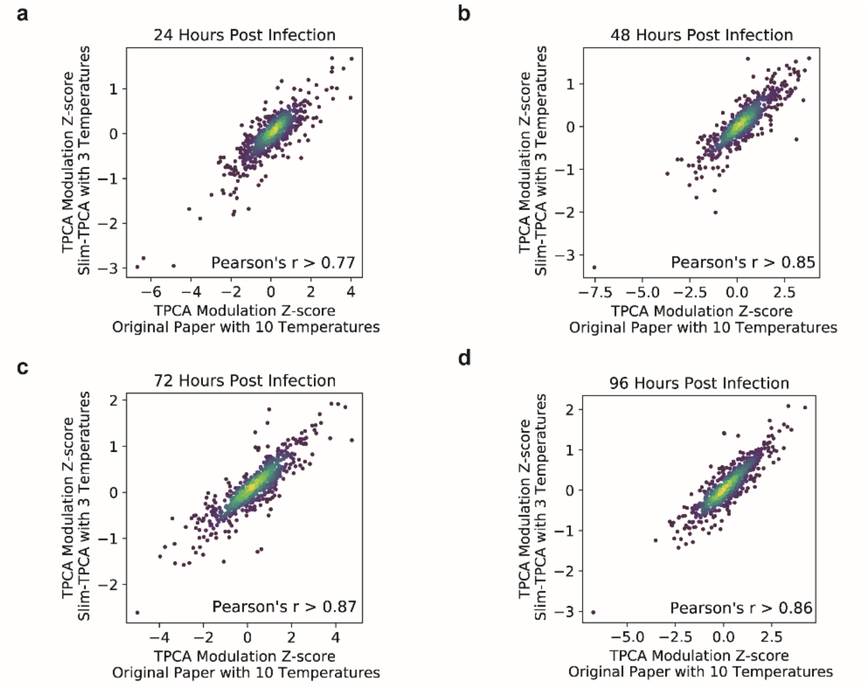

Supplementary Figure 5. Scatter plot of TPCA modulation z-scores. (**a-d**) The TPCA modulation z-scores obtained using three temperature points maintained a good correlation with those obtained by the conventional TPCA method. The four subplots correspond to complex TPCA modulation signatures at 24 h, 48 h, 72 h and 96 h post-infection, respectively.

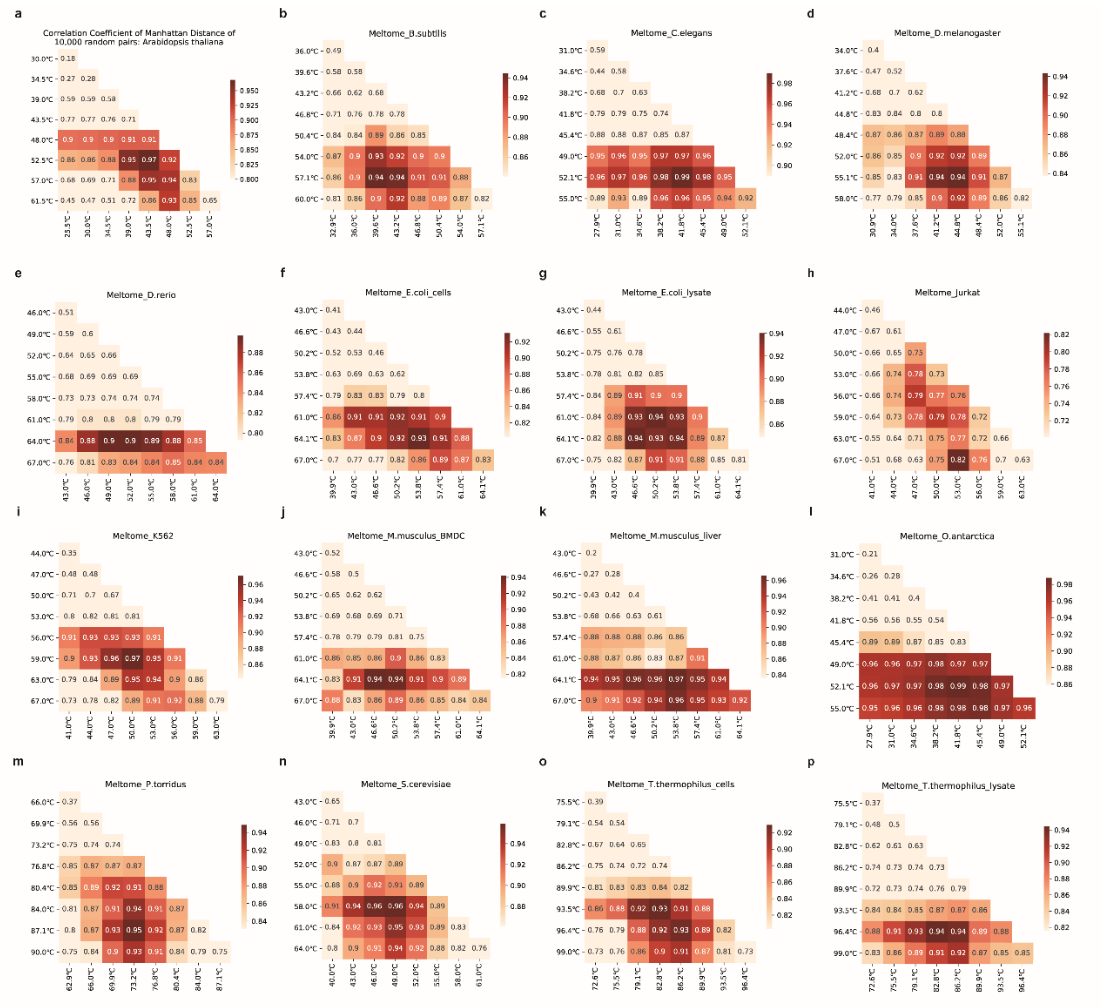

Supplementary Figure 6. Heatmap of correlation between Manhattan distances of 100,000 random protein pairs computed with 3 and 10 temperature points in different species. Data were obtained from studies on A. thaliana and from the Meltome database. These results suggest the framework proposed in this work could also be adopted for other species. (a) A. thaliana. (b) B. subtilis. (c) C. elegans. (d) D.melanogaster. (e) D. rerio. (f) E.coli_cells. (g) E.coli_lysate. (h) Jurkat. (i) K562. (j) M.musculus_BMDC. (k) M.musculus_liver. (l) O.antarctica. (m) P.torridus. (n) S.cerevisiae. (o) T.thermophilus_cells. (p) T.thermophilus _lysate.

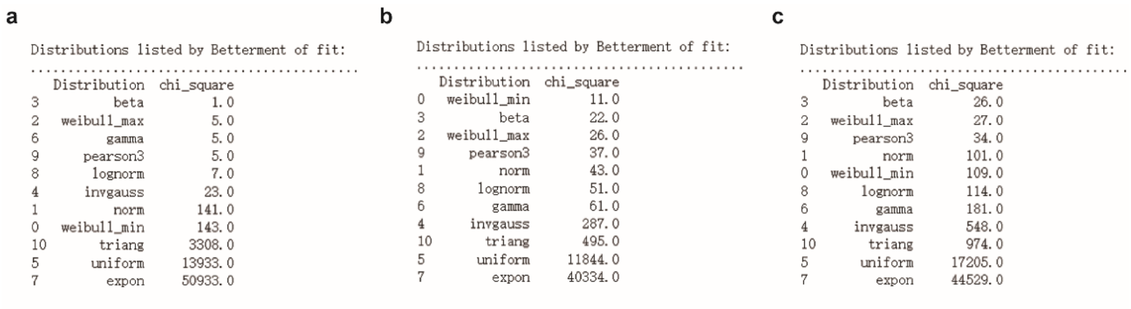

a

Distributions listed by Betterment of fit:

	Distribution	chi_square
3	beta	1.0
2	weibull_max	5.0
6	gamma	5.0
9	pearson3	5.0
8	lognorm	7.0
4	invgauss	23.0
1	norm	141.0
0	weibull_min	143.0
10	triang	3308.0
5	uniform	13933.0
7	expon	50933.0

b

Distributions listed by Betterment of fit:

	Distribution	chi_square
0	weibull_min	11.0
3	beta	22.0
2	weibull_max	26.0
9	pearson3	37.0
1	norm	43.0
8	lognorm	51.0
6	gamma	61.0
4	invgauss	287.0
10	triang	495.0
5	uniform	11844.0
7	expon	40334.0

c

Distributions listed by Betterment of fit:

	Distribution	chi_square
3	beta	26.0
2	weibull_max	27.0
9	pearson3	34.0
1	norm	101.0
0	weibull_min	109.0
8	lognorm	114.0
6	gamma	181.0
4	invgauss	548.0
10	triang	974.0
5	uniform	17205.0
7	expon	44529.0

Supplementary Figure 7. The beta distribution fits the distances obtained by the bootstrapping algorithm well in the chi-square test. (**a-c**) The results of using 500 complexes with different distances and various distributions to approximate the distances of 100,000 complexes. The 3 subplots correspond to the fit of the average distance, change in absolute distance, and change in relative distance, respectively. The Beta distribution performs well in all three approximations of distance.

Supplementary Figure 16-23

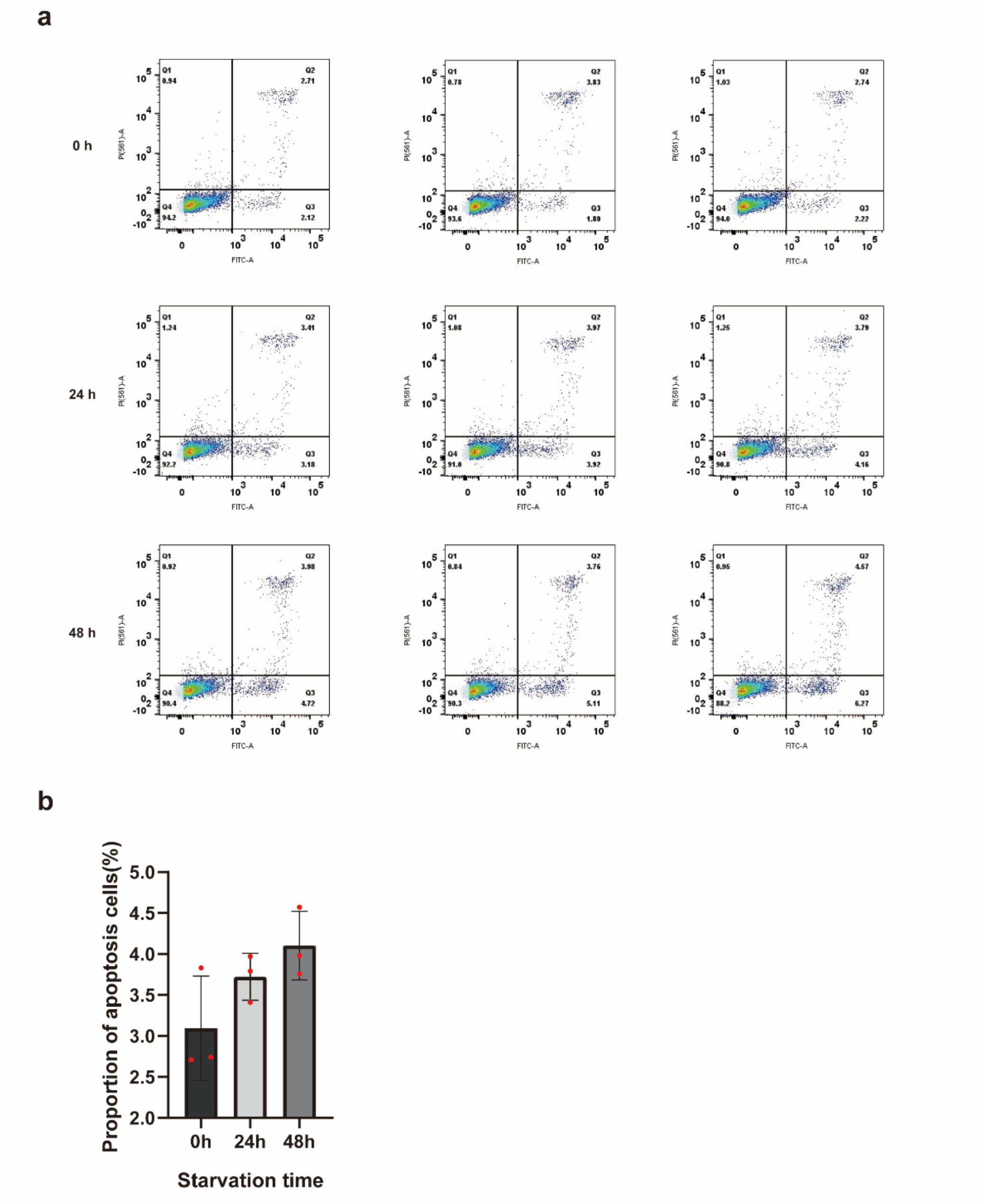

Supplementary Figure 16. FACS detect K562 cells apoptosis under glucose deprivation. (**a**) FACS analysis of K562 cells under glucose deprivation under 0th,24th and 48th (n = 3 independent technical experiments). (**b**) proportion of apoptosis cells under glucose deprivation under 0th,24th and 48th. Data are presented as mean values +/- SD. Source data are provided as a Source Data file.

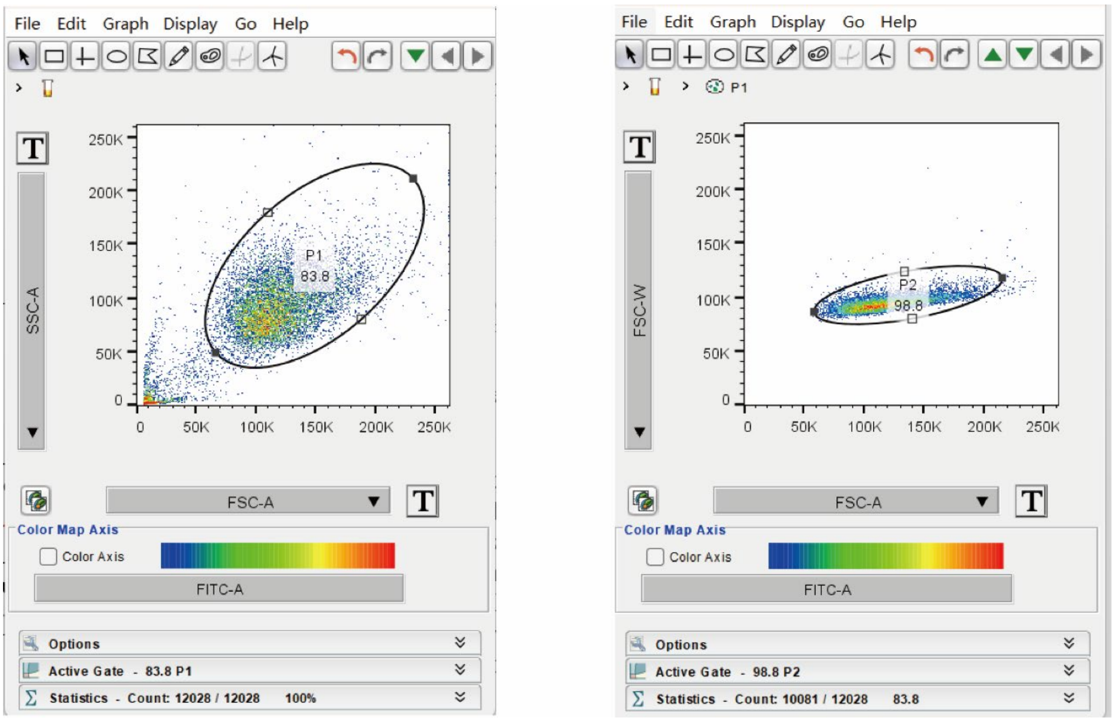

Supplementary Figure 17. FACS sequential gating/sorting strategies (correspond to Supplementary Figure 16). (**a**) The main cells are circled by FSC-A and SSC-A to exclude dead cells. (**b**) The main cells are circled by FSC-A and FSC-W to exclude the sticky cells.

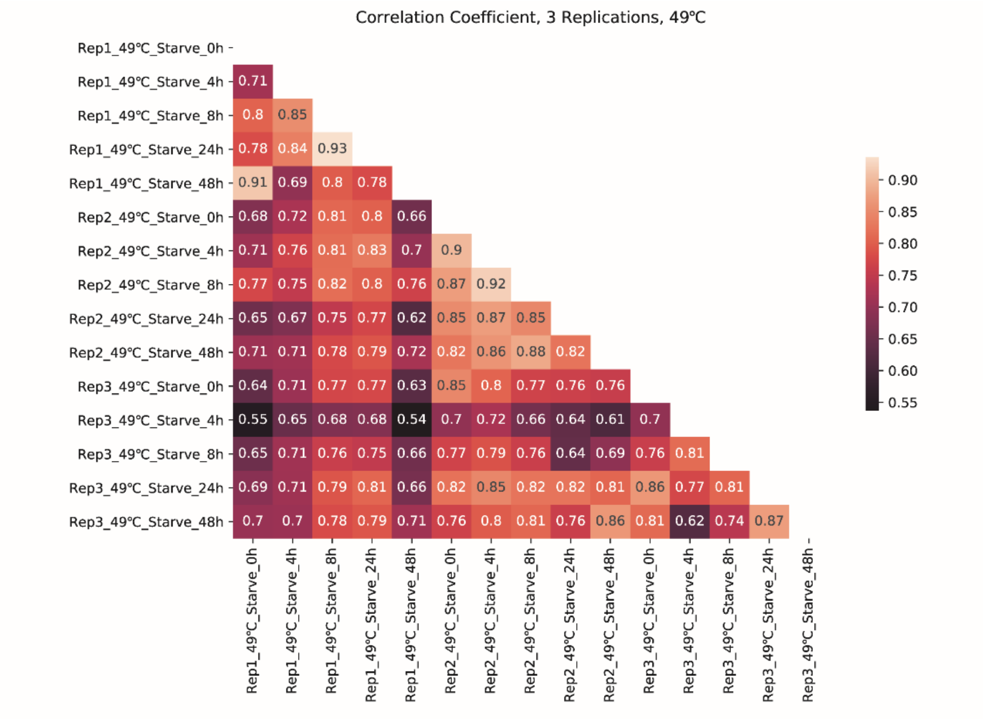

Supplementary Figure 18. Correlation heatmap of soluble fractions in data from repeated experiments at 49°C. The soluble fractions of proteins at the same 49°C and under different starvation conditions maintained a good correlation across the 3 replicates (n=5813 proteins).

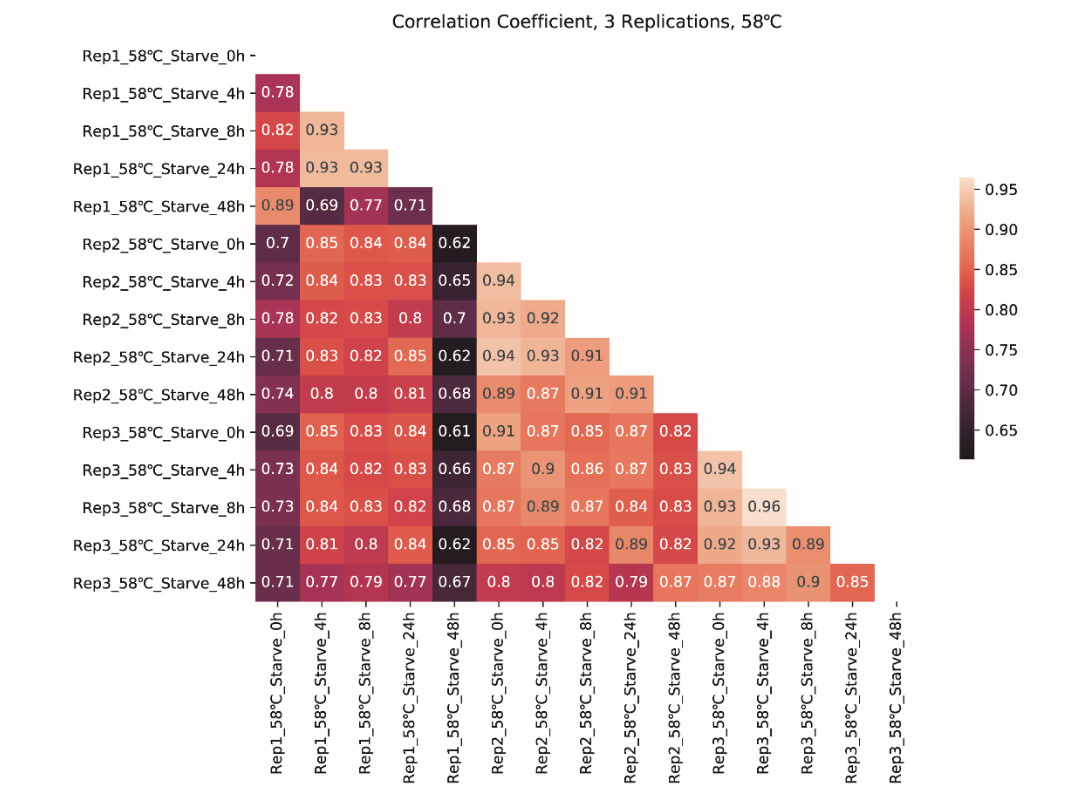

Supplementary Figure 19. Correlation heatmap of soluble fractions in data from repeated experiments at 58°C. The soluble fractions of proteins at the same 58°C and under different starvation conditions maintained a good correlation across the 3 replicates (n=5813 proteins).

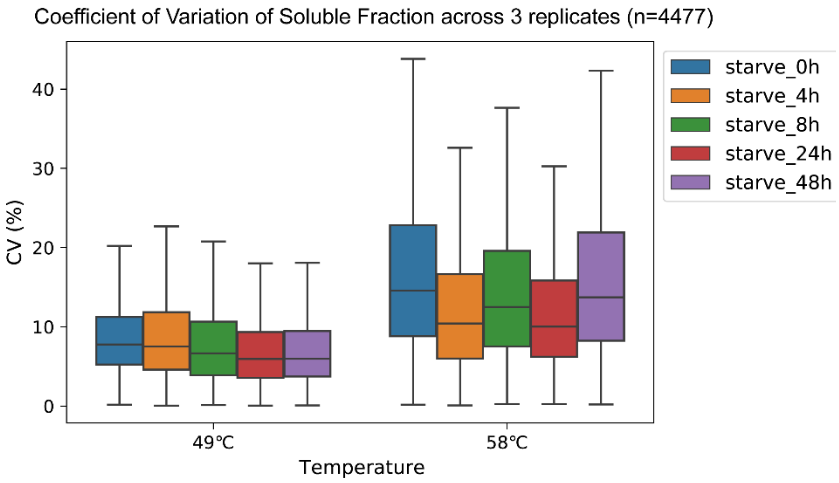

Supplementary Figure 20. Coefficient of variation of proteomic soluble fraction across 3 biological replicates. In a set of TMT16 experiments, 15 channels corresponded to different experimental conditions, i.e., combinations of 3 temperatures (37, 49, 58°C) and 5 cell states (0, 4, 8, 24, 48h after glucose deprivation). The coefficient of variation at 58°C is greater than that at 49°C due to overall lower protein abundance, but is well within acceptability (n=5813 proteins). Data are presented as mean values +/- SEM.

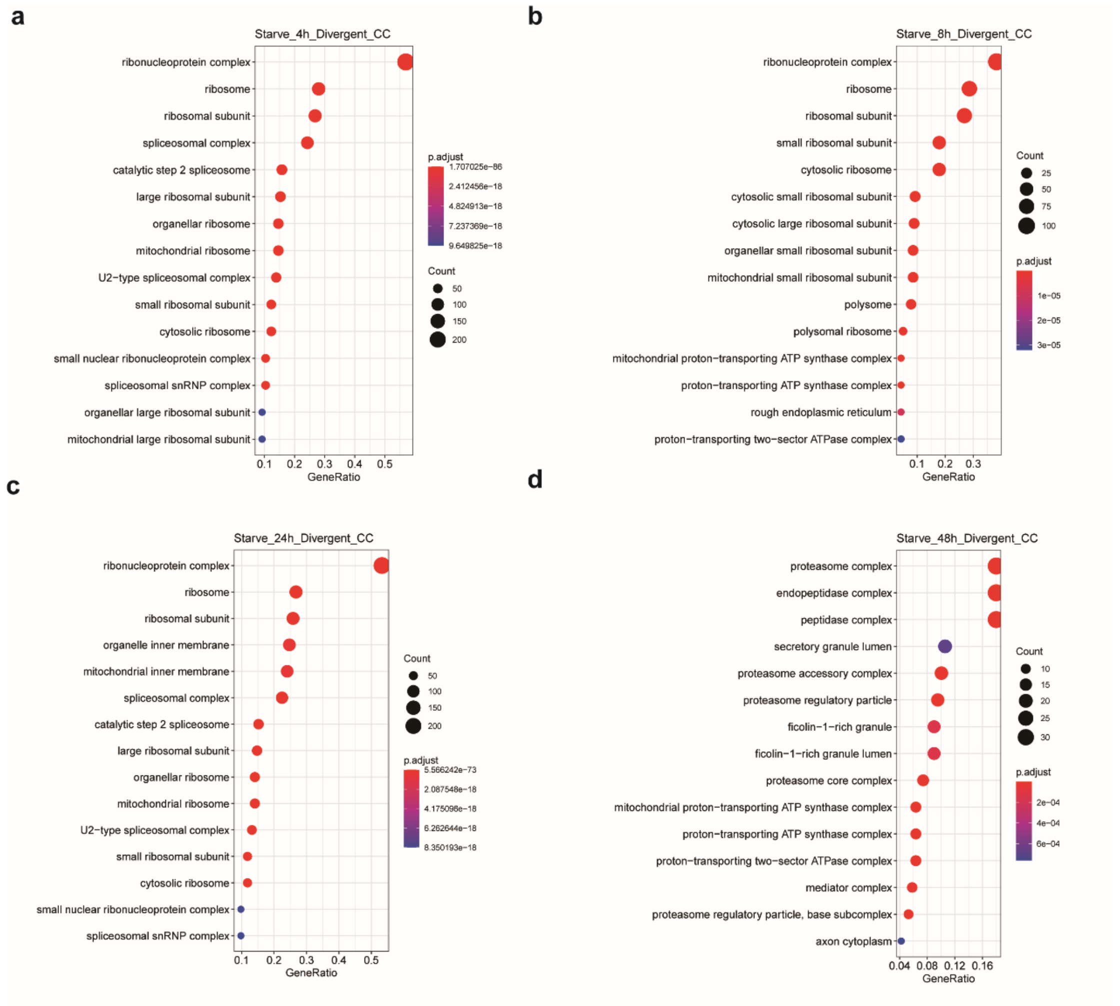

Supplementary Figure 21. Gene Ontology enrichment results for proteins in complexes significantly divergent after glucose deprivation. (**a**) Gene ontology enrichment analysis of proteins in significantly divergent complexes is performed for cells harvested after 4 hours of glucose deprivation. (**b**) Gene ontology enrichment analysis of proteins in significantly divergent complexes is performed for cells harvested after 8 hours of glucose deprivation. (**c**) Gene ontology enrichment analysis of proteins in significantly divergent complexes is performed for cells harvested after 24 hours of glucose deprivation. (**d**) Gene ontology enrichment analysis of proteins in significantly divergent complexes is performed for cells harvested after 48 hours of glucose deprivation.

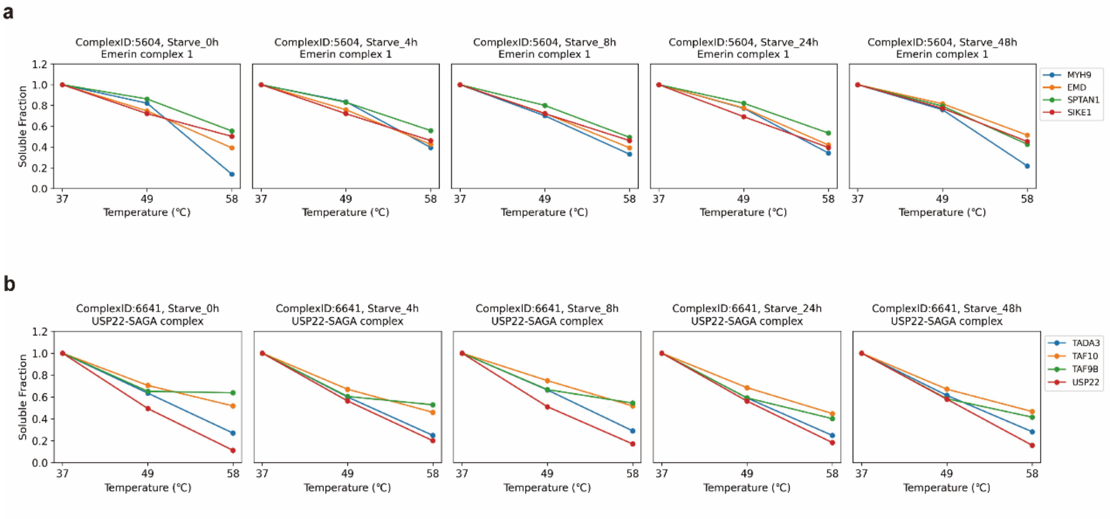

Supplementary Figure 22. TPCA profiling to K562 cells under glucose deprivation. (a-b) TPCA profile of two protein complexes Emerin complex 1 and USP22-SAGA complex at different time point of glucose deprivation.

Supplementary Methods

(1) Flow cytometry detection of apoptosis.

Cell apoptosis was detected using Annexin V-FITC Apoptosis Detection Kit (Beyotime, China). K562 cells were harvested and washed with ice-cold PBS twice prior resuspended in 1X annexin-binding buffer at 5 million cells per tube. Cells were then stained with FITC Annexin V and PI solution protecting from light at room temperature for 15 minutes and analyzed by flow cytometry (BD FACSCanto SORP) as soon as possible.

(2) Flow cytometry data analysis

Flow cytometry data was analyzed by software FlowJo_v10.8.1. Gating strategy: The gating strategy of the initial FSC/SSC gate ensured that most of the cell debris, bubbles, and laser noise interference (all in the FSC-low region) were excluded from the analysis area. The unstained were double-negative cells, PI-only staining resulted in the expected PI-staining positive cells, Annexin V-FITC-only staining resulted in the expected Annexin V-FITC-staining positive cells, Annexin V-FITC and PI double staining resulted in the expected Annexin V-FITC-only positive apoptotic cells and double-positive necrotic cells. The FACS data generated in this study are provided in the Source Data file.
