# Supplementary material for: Improved in situ characterization of protein complex dynamics at scale with thermal proximity co-aggregation
Source: Nat Commun. 2023 Nov 24;14:7697. doi: 10.1038/s41467-023-43526-2 (PMC10673876; doi:10.1038/s41467-023-43526-2)
Supplement: Supplementary file 3 — Description of Additional Supplementary Files [file 41467_2023_43526_MOESM3_ESM.pdf]

Description of Additional Supplementary Files

File name: Supplementary Data 1
Description: Three independent biological proteomic data for glucose deprivation in K562 cell. Related to Figure 5 and Figure 6. **Sheet 1**: Replication 1; **Sheet 2**: Replication 2; **Sheet 3**: Replication 3.

File name: Supplementary Data 2
Description: Data from the previous TPCA proof-of-concept paper. Related to Figure 1b-d, Figure 2a-d, Figure 3, Figure 4a-e, Supplementary Fig. 1-5.
Sheet 1: Protein pairs reported by previous papers. Information on proteins pairs was used to calculate the AUC (Area Under Curve) of ROC (Receiver Operating Characteristic) curve.
Sheet 2: Protein complexes reported by CORUM database. The information on the complexes was used as the reference for the TPCA analyses.
Sheet 3: Cell lysate data at 10 temperature points (37°C, 40°C, 43°C, 46°C, 49°C, 52°C, 55°C, 58°C, 61°C, 64°C) from the previous TPCA paper.
Sheet 4: Cell lysate data at 4 temperature points (37°C, 46°C, 55°C, 61°C) from the previous TPCA paper.
Sheet 5: Cell lysate data at 3 temperature points (37°C, 49°C, 58°C) from the previous TPCA paper.
Sheet 6: Intact cell data at 10 temperature points (37°C, 40°C, 43°C, 46°C, 49°C, 52°C, 55°C, 58°C, 61°C, 64°C) from the previous TPCA paper.
Sheet 7: Intact cell data at 4 temperature points (37°C, 46°C, 55°C, 61°C) from the previous TPCA paper.
Sheet 8: Intact cell data at 3 temperature points from the previous TPCA paper.
Sheet 9: DMSO-treated data at 10 temperature points from the previous TPCA paper.
Sheet 10: DMSO-treated data at 4 temperature points from the previous TPCA paper.
Sheet 11: DMSO-treated data at 3 temperature points from the previous TPCA paper.
Sheet 12: MTX-treated data at 10 temperature points from the previous TPCA paper.
Sheet 13: MTX-treated data at 4 temperature points from the previous TPCA paper.
Sheet 14: MTX-treated data at 3 temperature points from the previous TPCA paper.

File name: Supplementary Data 3
Description: Data from the previous paper on human cytomegalovirus (HCMV) infection published by Hashimoto et al. Related to Figure 2e, Figure 4f-h.
Sheet 1: Pre-infection data at 10 temperature points (36.9°C, 40.2°C, 43.9°C, 46.6°C, 48.6°C, 52.7°C, 55.3°C, 58.5°C, 61.2°C, 64°C).
Sheet 2: 24 hours post-infection data at 10 temperature points.
Sheet 3: 48 hours post-infection data at 10 temperature points.
Sheet 4: 72 hours post-infection data at 10 temperature points.
Sheet 5: 96 hours post-infection data at 10 temperature points.
Sheet 6: Pre-infection data at 4 temperature points (36.9°C, 46.6°C, 55.3°C, and 61.2°C).

Sheet 7: 24 hours post-infection data at 4 temperature points.
Sheet 8: 48 hours post-infection data at 4 temperature points.
Sheet 9: 72 hours post-infection data at 4 temperature points.
Sheet 10: 96 hours post-infection data at 4 temperature points.
Sheet 11: Pre-infection data at 3 temperature points (36.9°C, 48.6°C, 58.5°C).
Sheet 12: 24 hours post-infection data at 3 temperature points.
Sheet 13: 48 hours post-infection data at 3 temperature points.
Sheet 14: 72 hours post-infection data at 3 temperature points.
Sheet 15: 96 hours post-infection data at 3 temperature points.

File name: Supplementary Data 4
Description: Data from 3 replicate glucose deprivation experiments. Related to Figure 5, Figure 6a. Supplementary Fig. 18-22.
Sheet 1: Original proteomics profiling results from first replicate experiment.
Sheet 2: Original proteomics profiling results from second replicate experiment.
Sheet 3: Original proteomics profiling results from third replicate experiment.
Sheet 4: Normalized proteomics results from first replicate experiment.
Sheet 5: Normalized proteomics results from second replicate experiment.
Sheet 6: Normalized proteomics results from third replicate experiment.
Sheet 7: The processed results obtained by filtering and summarising the data from the three replicate experiments.
