# Supplementary material for: Improved in situ characterization of protein complex dynamics at scale with thermal proximity co-aggregation
Source: Nat Commun. 2023 Nov 24;14:7697. doi: 10.1038/s41467-023-43526-2 (PMC10673876; doi:10.1038/s41467-023-43526-2)
Supplement: Supplementary file 8 — Reporting Summary [file 41467_2023_43526_MOESM8_ESM.pdf]

nature portfolio

Corresponding author(s): Chris Soon Heng TAN

Last updated by author(s): Oct 27, 2023

Reporting Summary

Nature Portfolio wishes to improve the reproducibility of the work that we publish. This form provides structure for consistency and transparency in reporting. For further information on Nature Portfolio policies, see our Editorial Policies and the Editorial Policy Checklist.

Statistics

For all statistical analyses, confirm that the following items are present in the figure legend, table legend, main text, or Methods section.

n/a	Confirmed	
☐	☒	The exact sample size (*n*) for each experimental group/condition, given as a discrete number and unit of measurement
☐	☒	A statement on whether measurements were taken from distinct samples or whether the same sample was measured repeatedly
☐	☒	The statistical test(s) used AND whether they are one- or two-sided<br>*Only common tests should be described solely by name; describe more complex techniques in the Methods section.*
☒	☐	A description of all covariates tested
☐	☒	A description of any assumptions or corrections, such as tests of normality and adjustment for multiple comparisons
☐	☒	A full description of the statistical parameters including central tendency (e.g. means) or other basic estimates (e.g. regression coefficient) AND variation (e.g. standard deviation) or associated estimates of uncertainty (e.g. confidence intervals)
☐	☒	For null hypothesis testing, the test statistic (e.g. *F*, *t*, *r*) with confidence intervals, effect sizes, degrees of freedom and *P* value noted<br>*Give P values as exact values whenever suitable.*
☒	☐	For Bayesian analysis, information on the choice of priors and Markov chain Monte Carlo settings
☒	☐	For hierarchical and complex designs, identification of the appropriate level for tests and full reporting of outcomes
☐	☒	Estimates of effect sizes (e.g. Cohen's *d*, Pearson's *r*), indicating how they were calculated

Our web collection on statistics for biologists contains articles on many of the points above.

Software and code

Policy information about availability of computer code

Data collection	All the mass spectrometry data were collected using Thermo Scientific Xcalibur software(version 4.1.50). All WB images were collected using Odyssey infrared scanner (LICOR Bioscience, Lincoln, NE, USA). All Luminescence data were collected using microplate reader (EnSpire) . All FACS data were collected by BD FACSCanto SORP.
Data analysis	Raw files were searched using Proteome Discoverer (PD) software (Version 2.4, Thermo Fisher Scientific) against the human proteome fasta database (Uniprot,20376 entries, downloaded on May 03, 2022).<br>Luminescence data was analyzed by GraphPad Prism(version 8.0.2).<br>Data analysis and visualization was programmed using python (version 3.7) and R (version 4.1). Packages involved in the analysis include numpy (version 1.24.3), pandas (version 1.5.3), matplotlib (version 3.7.1), scipy (version 1.10.1), seaborn (version 0.12.2), sklearn (version 1.2.2), re (version 2.2.1), statsmodels (version 0.13.5).<br>Molecular function and cellular compartment annotation were referred to clusterProfiler package (Version 3.16.1) in R and GO knowledgebase (Released at 2020-06-01, with 44,411 GO terms and 7,975,639 annotations; FDR<0.05).<br>The installation package of Slim-TPCA can be downloaded directly from PyPI (https://pypi.org/project/Slim-TPCA/). Package documentation can be acquired online via https://slim-tpca.readthedocs.io/en/latest/index.html. Testing data can be downloaded from GitHub(https://github.com/wangjun258/Slim_TPCA_examples).

For manuscripts utilizing custom algorithms or software that are central to the research but not yet described in published literature, software must be made available to editors and reviewers. We strongly encourage code deposition in a community repository (e.g. GitHub). See the Nature Portfolio guidelines for submitting code & software for further information.

Data

Policy information about availability of data

All manuscripts must include a data availability statement. This statement should provide the following information, where applicable:

- Accession codes, unique identifiers, or web links for publicly available datasets
- A description of any restrictions on data availability
- For clinical datasets or third party data, please ensure that the statement adheres to our policy

All the raw MS data have been deposited to the ProteomeXchange Consortium via the iProX partner repositor with the dataset identifier PXD040078.
Information on protein pair interactions was referred to iRefWeb database (version 13.0). Link: https://www.re3data.org/repository/r3d100012725
Protein complex information was referred to CORUM database (version 3.0). Link: http://mips.helmholtz-muenchen.de/corum
Molecular function and cellular compartment annotation were referred to clusterProfiler package (Version 3.16.1) in R and GO knowledgebase (Released at 2020-06-01, with 44,411 GO terms and 7,975,639 annotations; FDR<0.05).

Human research participants

Policy information about studies involving human research participants and Sex and Gender in Research.

Reporting on sex and gender	N/A
Population characteristics	N/A
Recruitment	N/A
Ethics oversight	N/A

Note that full information on the approval of the study protocol must also be provided in the manuscript.

Field-specific reporting

Please select the one below that is the best fit for your research. If you are not sure, read the appropriate sections before making your selection.

☒ Life sciences ☐ Behavioural & social sciences ☐ Ecological, evolutionary & environmental sciences

For a reference copy of the document with all sections, see nature.com/documents/nr-reporting-summary-flat.pdf

Life sciences study design

All studies must disclose on these points even when the disclosure is negative.

Sample size	Samples from three (n=3) biological replicate experiments (performed on cells on different days) were collected for analysis in this paper for the following reasons: 1. To assess quantitative accuracy and reproducibility of data obtained across three biological replicates; 2. To meet the requirements of basic statistical analysis (e.g. T-test); 3. To minimize idiosyncrasy pertaining to each biological experiment/sample. The sample size of proteins used for analysis (n=5813) in the glucose deprivation experiment is based on the data exclusion criteria in the later section.
Data exclusions	The glucose deprivation experiment was repeated 3 times. Proteins that were identified at least twice in the 3 experiments and have a total number of peptide-spectral matches (PSMs) greater than or equal to 3 were selected for the subsequent analysis. For data analysis, the soluble fraction at 49°C and 58°C for each protein was calculated using the abundance at 37°C as a standard. Proteins with soluble fractions above 1.2 at 49°C and 58°C were excluded because this is inconsistent with the nature of proteins decreasing in solubility with increasing temperature, which may be caused by re-solubilization.
Replication	All the mass spectrometry analysis were done with three biological replicates. For all the biological findings reported in this paper, all experiments were successfully replicated with the properly optimized condition. For western blot and imaging, the results were all repeated with at least three times (n>=3). For ATP measurement, the result were all repeated with at least three times (n>=3). For FACS measurement, the result were done with three technical replications (n=3). Attempts at all the replication were all successful.
Randomization	Randomization was not applicable to the study and experiment.
Blinding	Experiments were not operated blinded as it is not applicable.

Reporting for specific materials, systems and methods

We require information from authors about some types of materials, experimental systems and methods used in many studies. Here, indicate whether each material, system or method listed is relevant to your study. If you are not sure if a list item applies to your research, read the appropriate section before selecting a response.

Materials & experimental systems

n/a	Involved in the study
☐	☒ Antibodies
☐	☒ Eukaryotic cell lines
☒	☐ Palaeontology and archaeology
☒	☐ Animals and other organisms
☒	☐ Clinical data
☒	☐ Dual use research of concern

Methods

n/a	Involved in the study
☒	☐ ChIP-seq
☐	☒ Flow cytometry
☒	☐ MRI-based neuroimaging

Antibodies

Antibodies used	The primary antibodies used in this study were Phospho-Rb (Ser780) (CST,8180S,1:1000) , Phospho-Histone H3(Ser10) (CST,3377S,1:1000) , Phospho-CDK Substrate (CST,14371S,1:1000) , and β-Tubulin (CST,2128S,1:1000) ,GAPDH (Proteintech,10494-1-AP,1:5000), MYH9 (Proteintech,11128-1-AP, 1:10000), Emerin antibody (Proteintech, 10351-1-AP,1:5000), TAF9B antibody (Proteintech, 28713-1-AP,1:3000), TADA3L antibody (Proteintech, 10839-1-AP,1:4500). The secondary antibody HRP-labeled Goat Anti-Rabbit IgG(H+L) (Beyotime,A0208,1:2000), IPKine HRP, Mouse anti-Rabbit IgG LCS (Abbkine Scientific, A25022,1:2000), IPKine HRP, Goat Anti-Rabbit IgG HCS (Abbkine Scientific, A25222,1:2000).
Validation	1.Phospho-Rb (Ser780):CST,catalog number 8180S.(https://www.cellsignal.com/products/primary-antibodies/phospho-rb-ser780-d59b7-rabbit-mab/8180?site-search-type=Products&N=4294956287&Ntt=8180s&fromPage=plp&_requestid=370303)<br>2.Phospho-Histone H3(Ser10):CST,catalog number 3377S.(https://www.cellsignal.com/products/primary-antibodies/phospho-histone-h3-ser10-d2c8-xp-rabbit-mab/3377?site-search-type=Products&N=4294956287&Ntt=3377s&fromPage=plp&_requestid=370590)<br>3.Phospho-CDK Substrate:CST,catalog number 14371S. (https://www.cellsignal.com/products/primary-antibodies/phospho-cdk-substrate-ptpxk-d9v5n-rabbit-mab/14371?site-search-type=Products&N=4294956287&Ntt=14371s&fromPage=plp&_requestid=370703)<br>4.β-Tubulin:CST,catalog number 2128S.(https://www.cellsignal.com/products/primary-antibodies/b-tubulin-9f3-rabbit-mab/2128?site-search-type=Products&N=4294956287&Ntt=2128s&fromPage=plp&_requestid=370797)<br>5.GAPDH:Proteintech,catalog number 10494-1-AP.(https://www.ptgcn.com/products/GAPDH-Antibody-10494-1-AP.htm)<br>6.HRP-labeled Goat Anti-Rabbit IgG(H+L):Beyotime,catalog number A0208.(https://abclonal.com/catalog-antibodies/Bcl2RabbitpAb/A0208)<br>7.Emerin:Proteintech,catalog number 10351-1-AP.(https://www.ptgcn.com/products/EMD-Antibody-10351-1-AP.htm)<br>8.MYH9:Proteintech,catalog number 11128-1-AP.(https://www.ptgcn.com/products/MYH9-Antibody-11128-1-AP.htm)<br>9.TAF9B :Proteintech,catalog number 28713-1-AP.(https://www.ptgcn.com/products/TAF9B-Antibody-28713-1-AP.htm)<br>10.TADA3L :Proteintech,catalog number 10839-1-AP.(https://www.ptgcn.com/products/TADA3L-Antibody-10839-1-AP.htm)<br>11.IPKine HRP, Mouse anti-Rabbit IgG LCS :Abbkine Scientific,catalog number A25022.(https://www.abbkine.com/product/ipkine-hrp-mouse-anti-rabbit-igg-lcs-a25022/)<br>12.IPKine HRP, Goat Anti-Rabbit IgG HCS :Abbkine Scientific,catalog number A25222.(https://www.abbkine.com/product/ipkine-hrp-goat-anti-rabbit-igg-hcs-a25222/)

Eukaryotic cell lines

Policy information about cell lines and Sex and Gender in Research

Cell line source(s)	K562 cell lines was purchased from American Type Culture Collection.
Authentication	Cell line from ATCC and JCRB was authenticated by STR profiling by the vendors.
Mycoplasma contamination	Cells were negative for mycoplasma contamination.
Commonly misidentified lines (See ICLAC register)	No commonly misidentified lines were used in the study.

Flow Cytometry

Plots

Confirm that:

☒ The axis labels state the marker and fluorochrome used (e.g. CD4-FITC).

☒ The axis scales are clearly visible. Include numbers along axes only for bottom left plot of group (a 'group' is an analysis of identical markers).

☐ All plots are contour plots with outliers or pseudocolor plots.

☒ A numerical value for number of cells or percentage (with statistics) is provided.

Methodology

Sample preparation	Cell apoptosis was detected using Annexin V-FITC Apoptosis Detection Kit (Beyotime, China). K562 cells under glucose deprivation under 0th,24th and 48th were harvested and washed with ice-cold PBS twice prior resuspended in 1X annexin-binding buffer at 0.5 million cells per tube. Cells were then stained with FITC Annexin V and PI solution protecting from light at room temperature for 15 minutes and analyzed by BD FACSCanto SORP as soon as possible.
Instrument	BD FACSCanto SORP
Software	FlowJo_v10.8.1
Cell population abundance	In the stage of sample preparation, cell population abundance is 0.5 million in order to make the cell concentration of the loading sample less than 1 million per milliliter.
Gating strategy	The unstained were double-negative cells, PI-only staining resulted in the expected PI-staining positive cells, Annexin V-FITC-only staining resulted in the expected Annexin V-FITC-staining positive cells, Annexin V-FITC and PI double staining resulted in the expected Annexin V-FITC-only positive apoptotic cells and double-positive necrotic cells.

☒ Tick this box to confirm that a figure exemplifying the gating strategy is provided in the Supplementary Information.
