# Supplementary material for: Improved in situ characterization of protein complex dynamics at scale with thermal proximity co-aggregation
Source: Nat Commun. 2023 Nov 24;14:7697. doi: 10.1038/s41467-023-43526-2 (PMC10673876; doi:10.1038/s41467-023-43526-2)
Supplement: Supplementary file 9 — Source data [file 41467_2023_43526_MOESM9_ESM.zip › Source Data/WB and Co-IP replications and quantification/Figuer.6c/Three replications.pptx]

## Slide 1
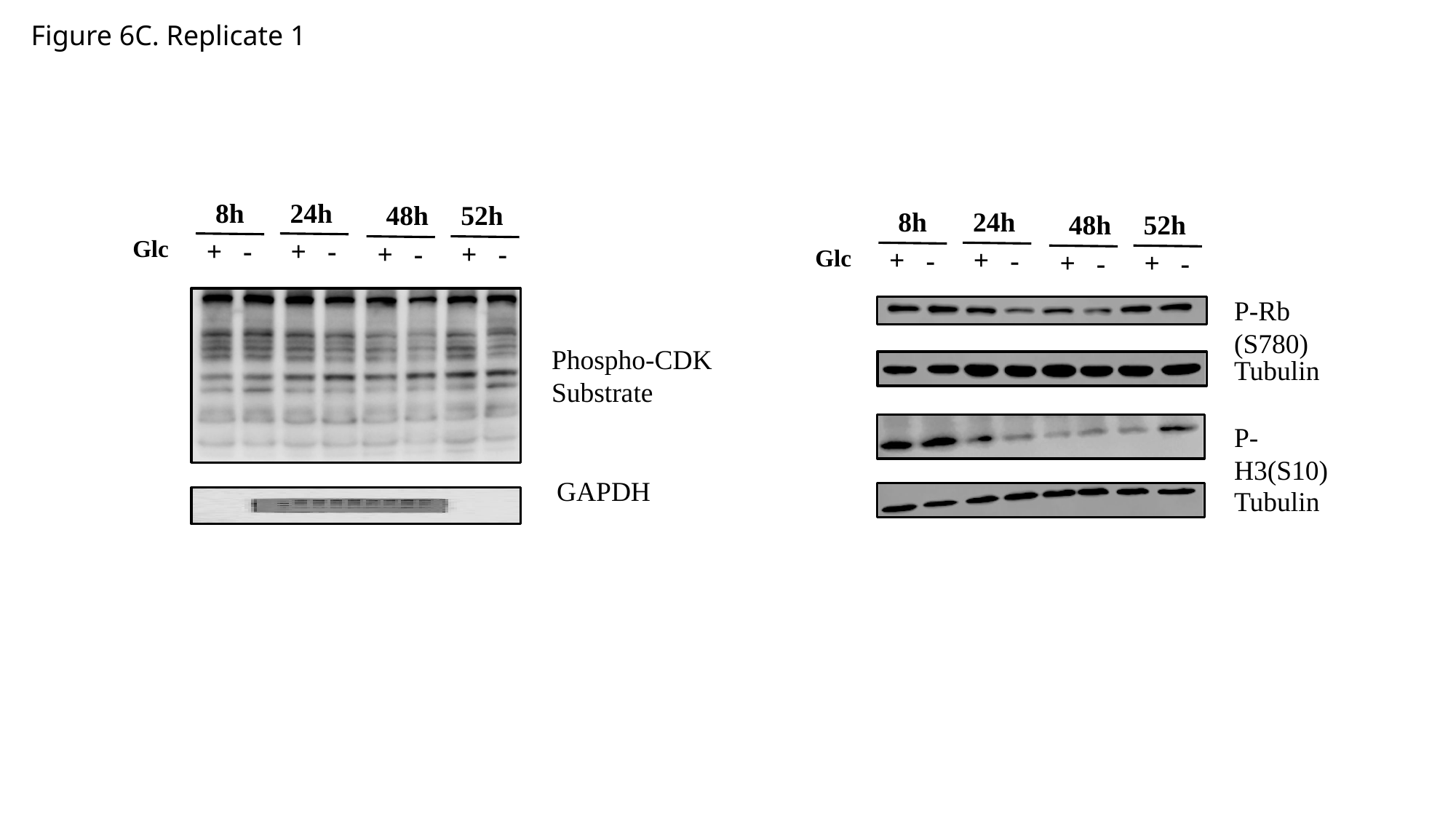

Figure 6C. Replicate 1
8h
24h
+
+
-
-
48h
52h
+
+
-
-
Glc
8h
24h
+
+
-
-
48h
52h
+
+
-
-
Glc
P-Rb (S780)
Tubulin
P-H3(S10)
Tubulin
Phospho-CDK Substrate
GAPDH

## Slide 2
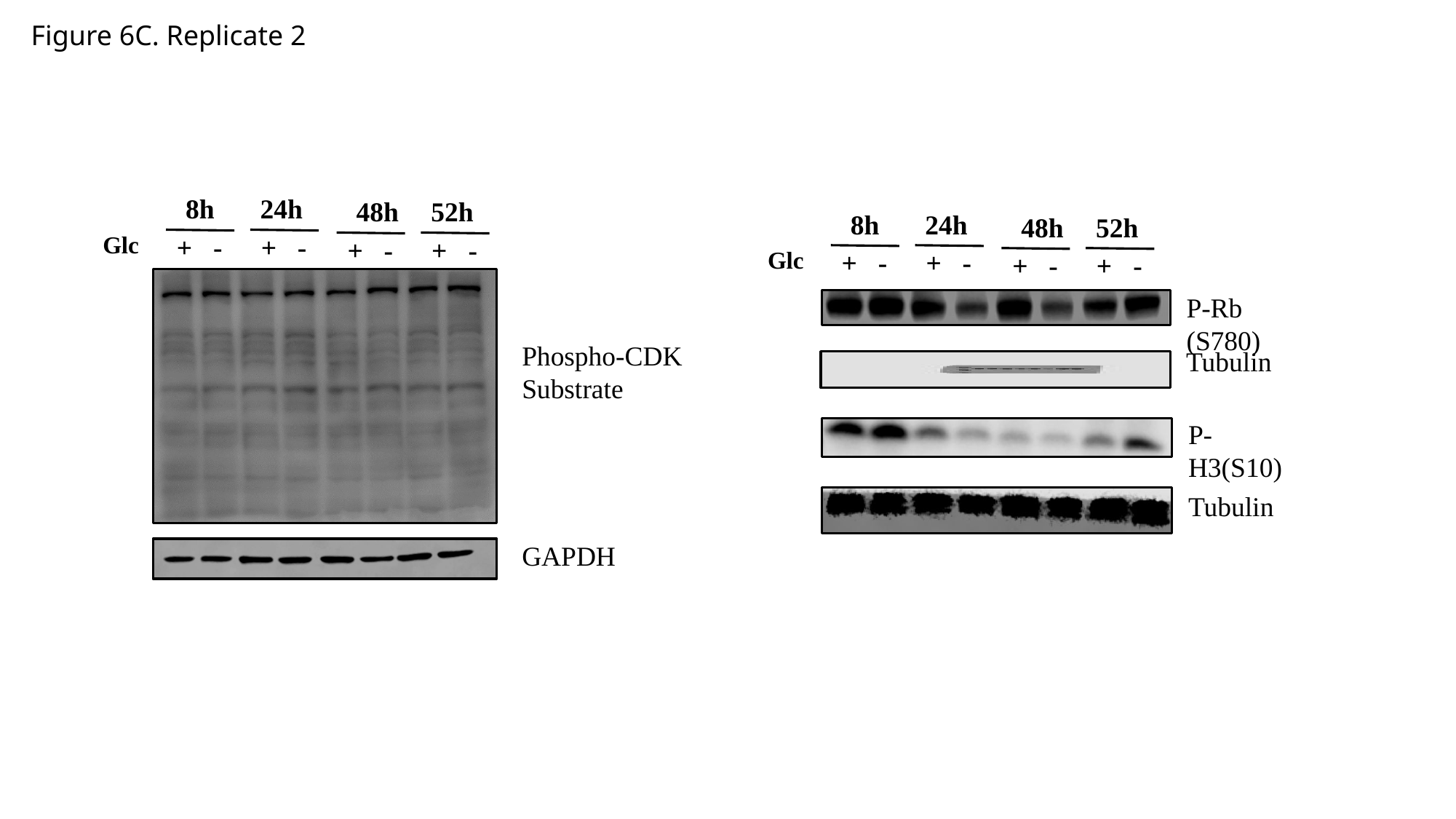

Figure 6C. Replicate 2
8h
24h
+
+
-
-
48h
52h
+
+
-
-
Glc
Phospho-CDK Substrate
GAPDH
8h
24h
+
+
-
-
48h
52h
+
+
-
-
Glc
P-Rb (S780)
Tubulin
P-H3(S10)
Tubulin

## Slide 3
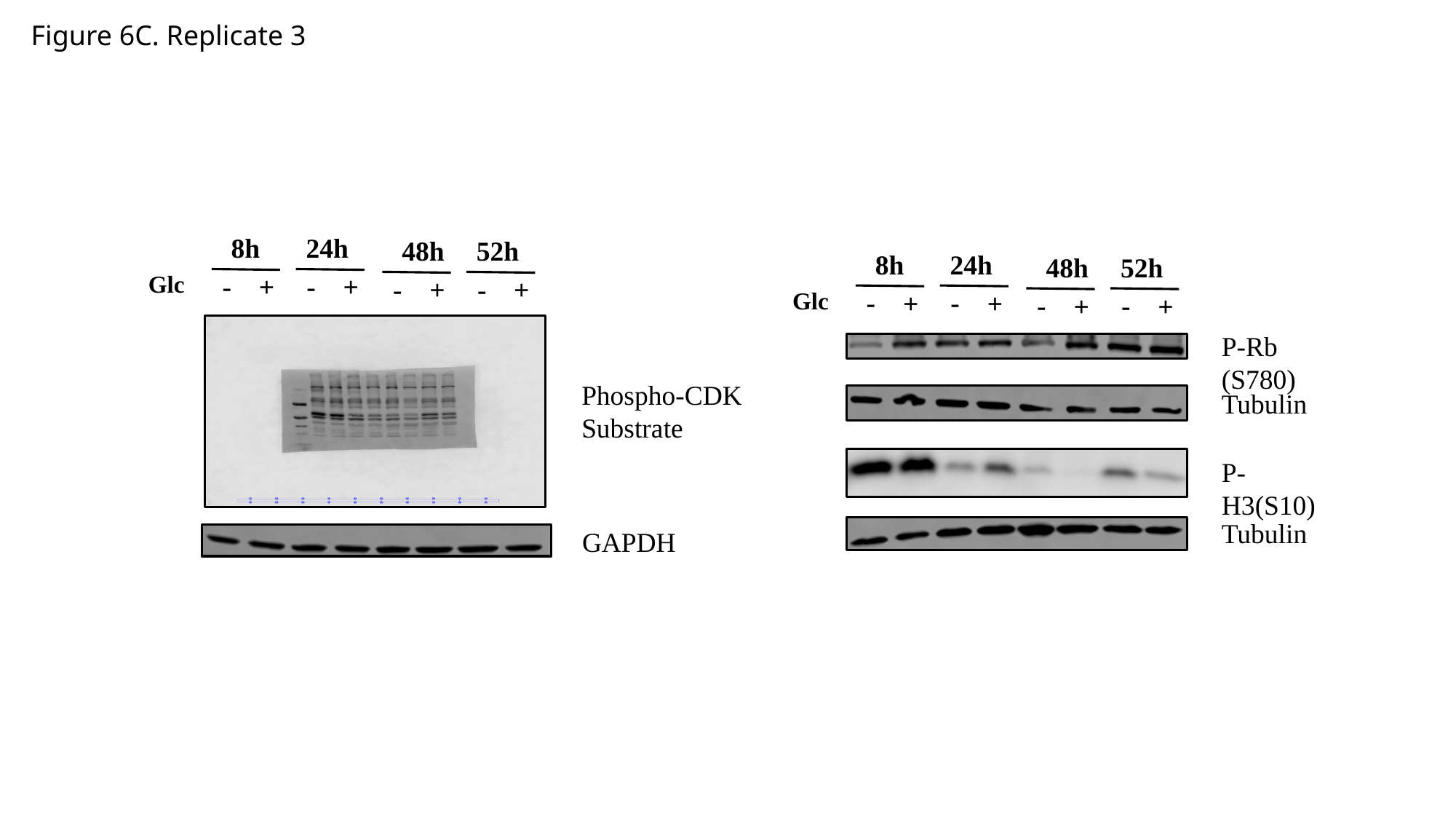

Figure 6C. Replicate 3
8h
24h
-
-
+
+
48h
52h
-
-
+
+
Glc
Phospho-CDK Substrate
GAPDH
8h
24h
-
-
+
+
48h
52h
-
-
+
+
Glc
P-Rb (S780)
Tubulin
P-H3(S10)
Tubulin
