# Supplementary material for: Improved in situ characterization of protein complex dynamics at scale with thermal proximity co-aggregation
Source: Nat Commun. 2023 Nov 24;14:7697. doi: 10.1038/s41467-023-43526-2 (PMC10673876; doi:10.1038/s41467-023-43526-2)
Supplement: Supplementary file 9 — Source data [file 41467_2023_43526_MOESM9_ESM.zip › Source Data/WB and Co-IP replications and quantification/Figuer.6e/Three replications.pptx]

## Slide 1
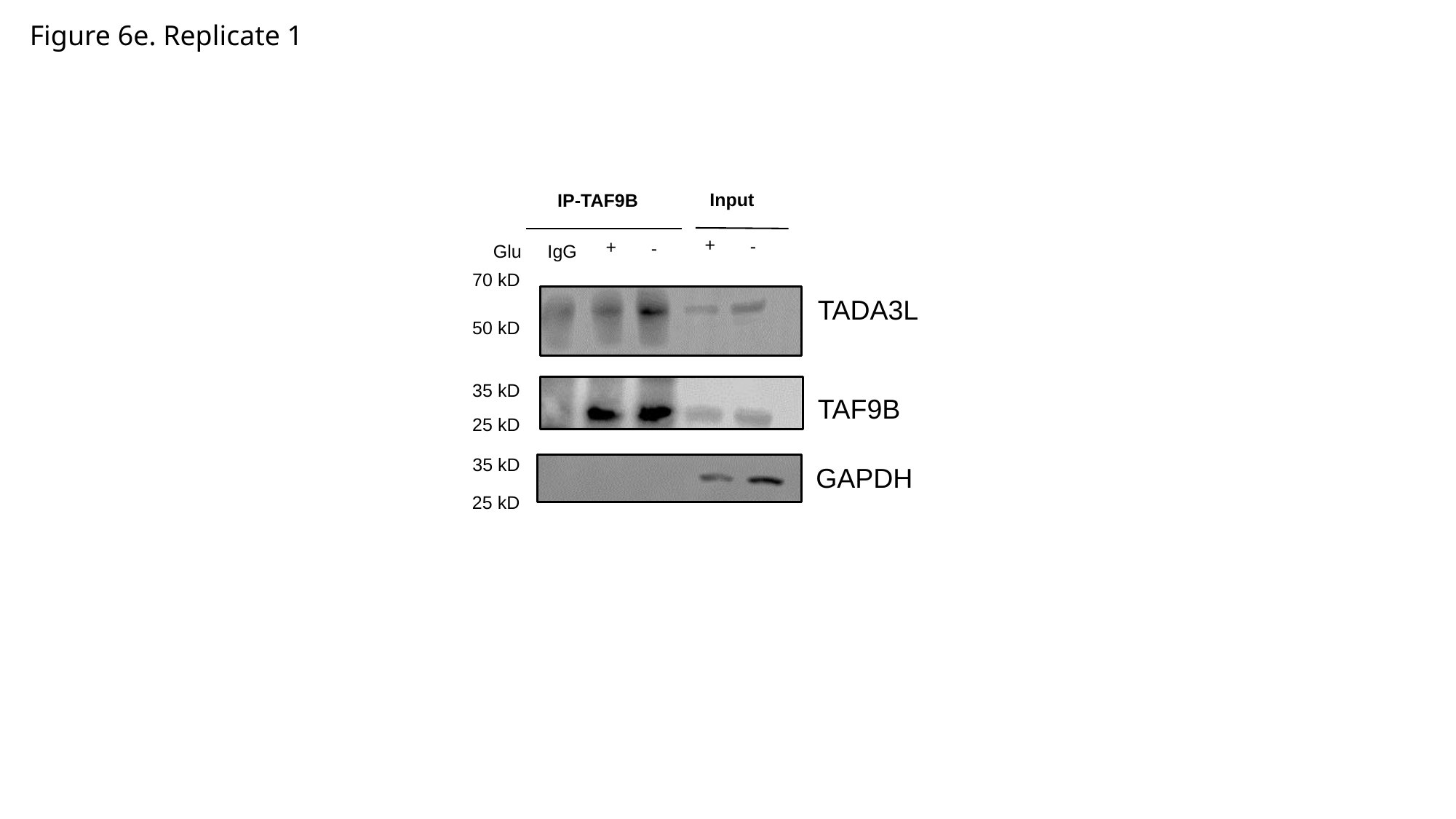

Figure 6e. Replicate 1
Input
IP-TAF9B
+
-
+
-
Glu
IgG
70 kD
TADA3L
50 kD
35 kD
TAF9B
25 kD
35 kD
GAPDH
25 kD

## Slide 2
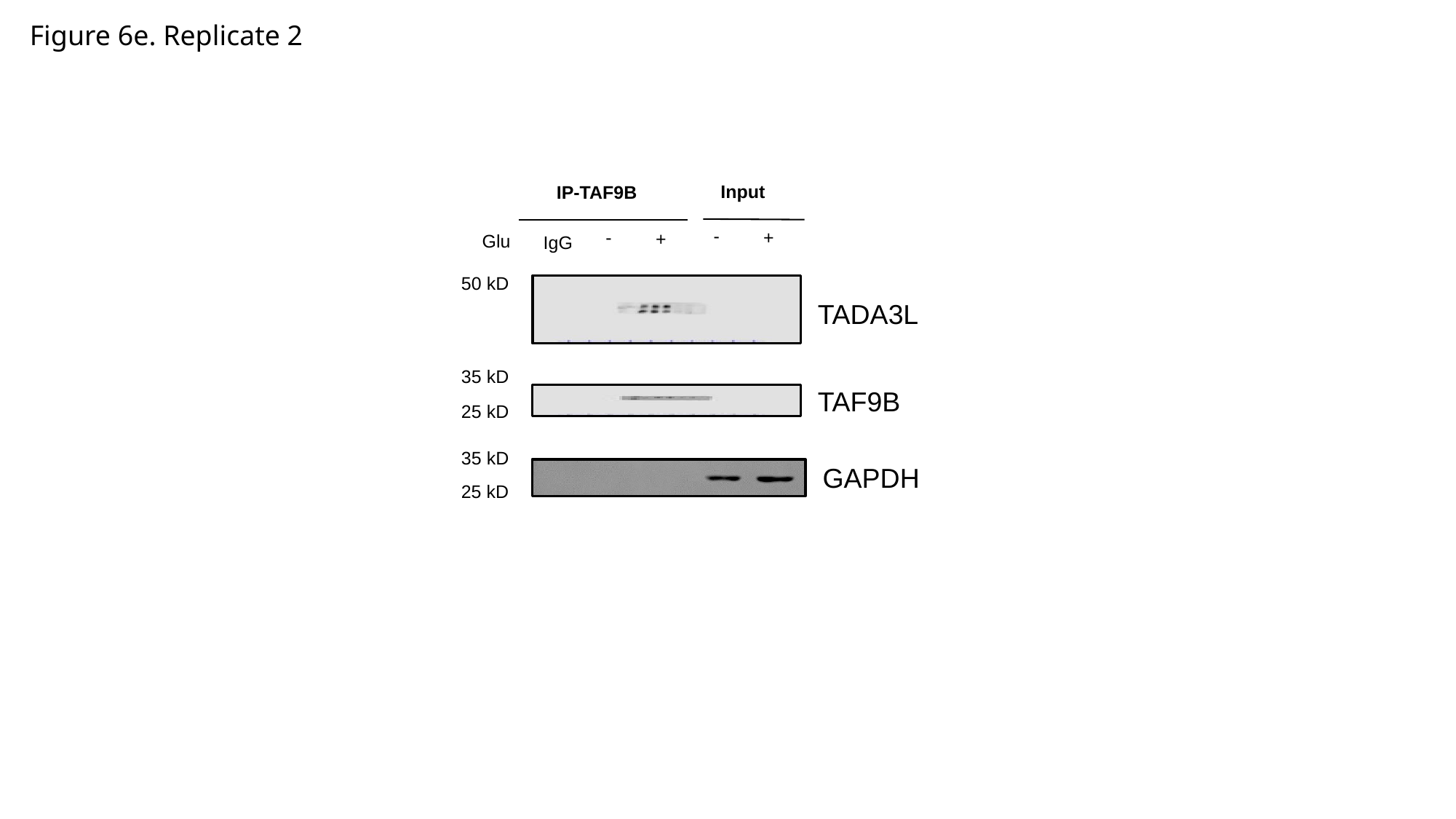

Figure 6e. Replicate 2
Input
IP-TAF9B
-
+
-
+
Glu
IgG
50 kD
TADA3L
35 kD
TAF9B
25 kD
35 kD
GAPDH
25 kD

## Slide 3
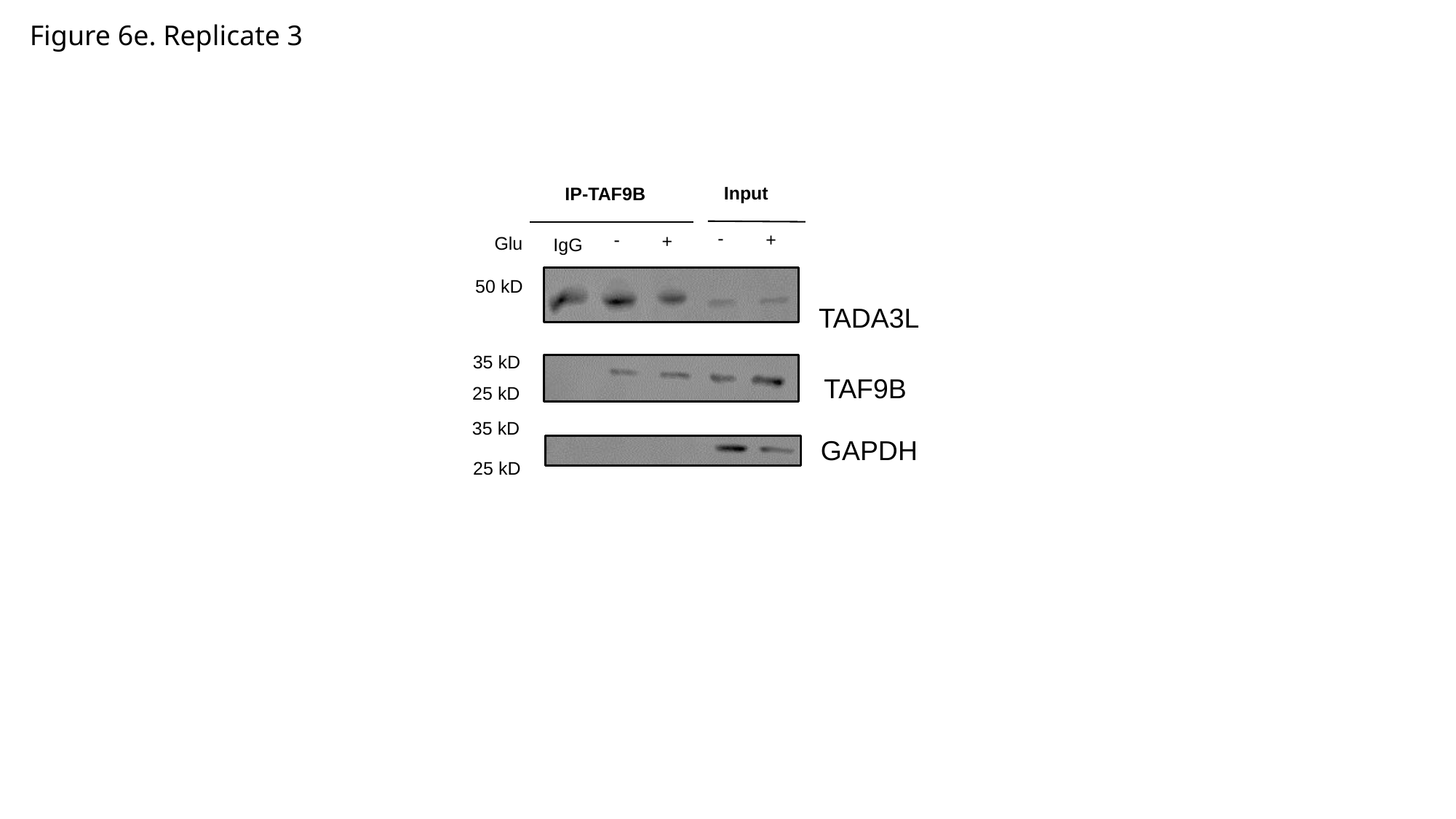

Figure 6e. Replicate 3
Input
IP-TAF9B
-
+
-
+
Glu
IgG
50 kD
TADA3L
35 kD
TAF9B
25 kD
35 kD
GAPDH
25 kD

## Slide 4
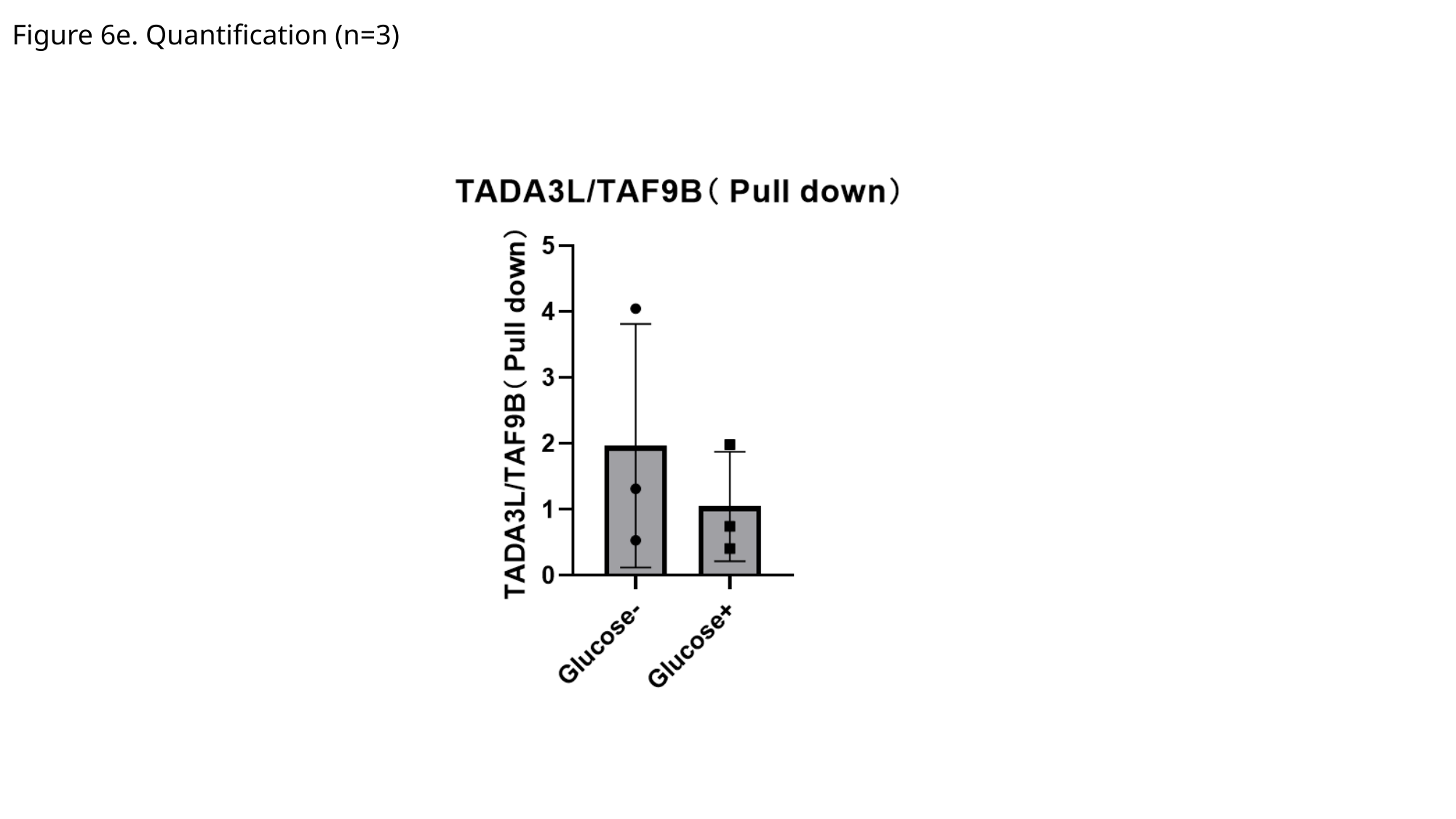

Figure 6e. Quantification (n=3)
