# Supplementary figures and images for: Improved in situ characterization of protein complex dynamics at scale with thermal proximity co-aggregation
Source: Nat Commun. 2023 Nov 24;14:7697. doi: 10.1038/s41467-023-43526-2 (PMC10673876; doi:10.1038/s41467-023-43526-2)

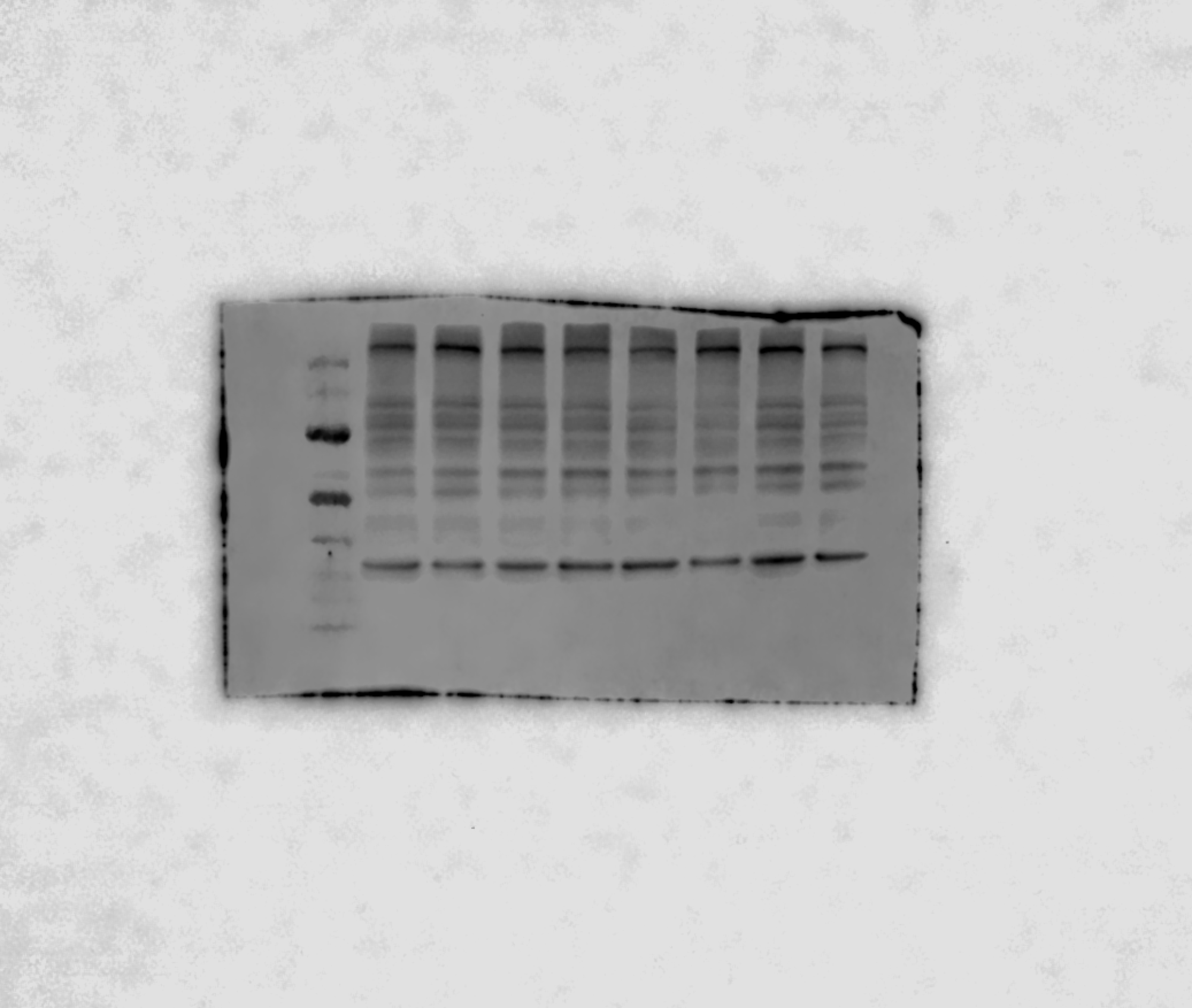

Supplement: Supplementary file 9 — Source data [file 41467_2023_43526_MOESM9_ESM.zip › Source Data/WB and Co-IP replications and quantification/Figuer.6c/replication_1/GAPDH.jpg.tif]

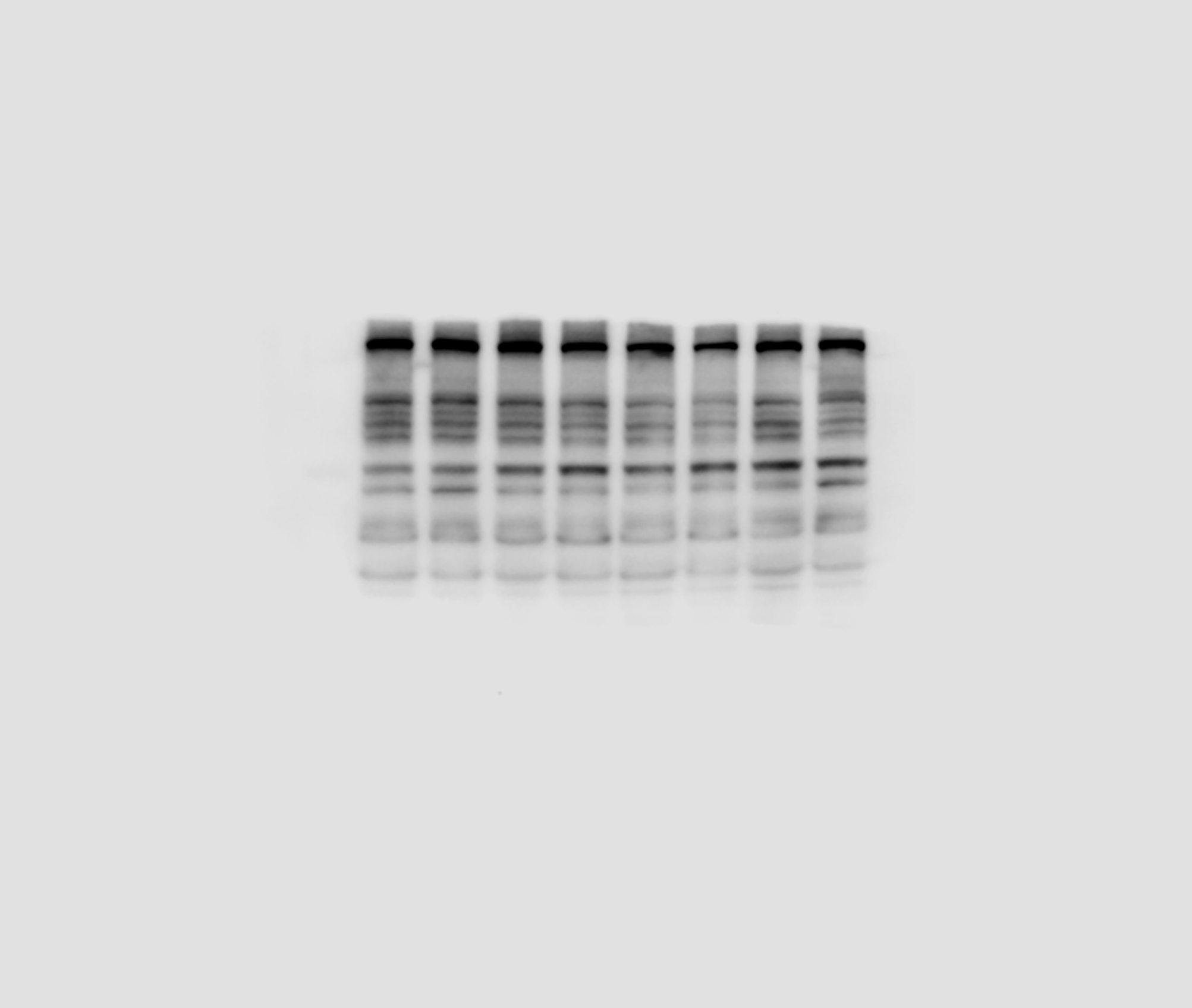

Supplement: Supplementary file 9 — Source data [file 41467_2023_43526_MOESM9_ESM.zip › Source Data/WB and Co-IP replications and quantification/Figuer.6c/replication_1/P-CDK.jpg.jpg]

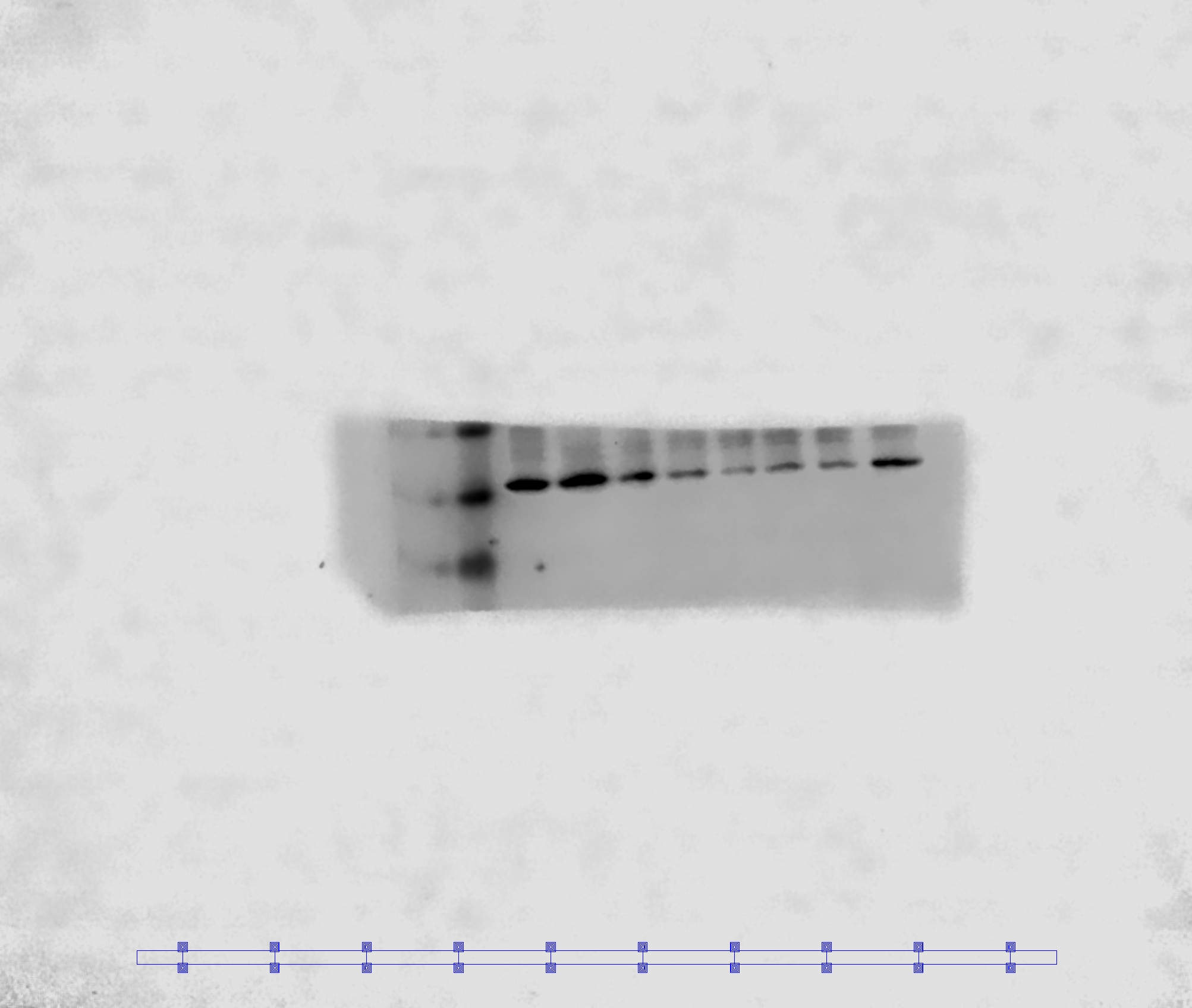

Supplement: Supplementary file 9 — Source data [file 41467_2023_43526_MOESM9_ESM.zip › Source Data/WB and Co-IP replications and quantification/Figuer.6c/replication_1/P-H3.jpg]

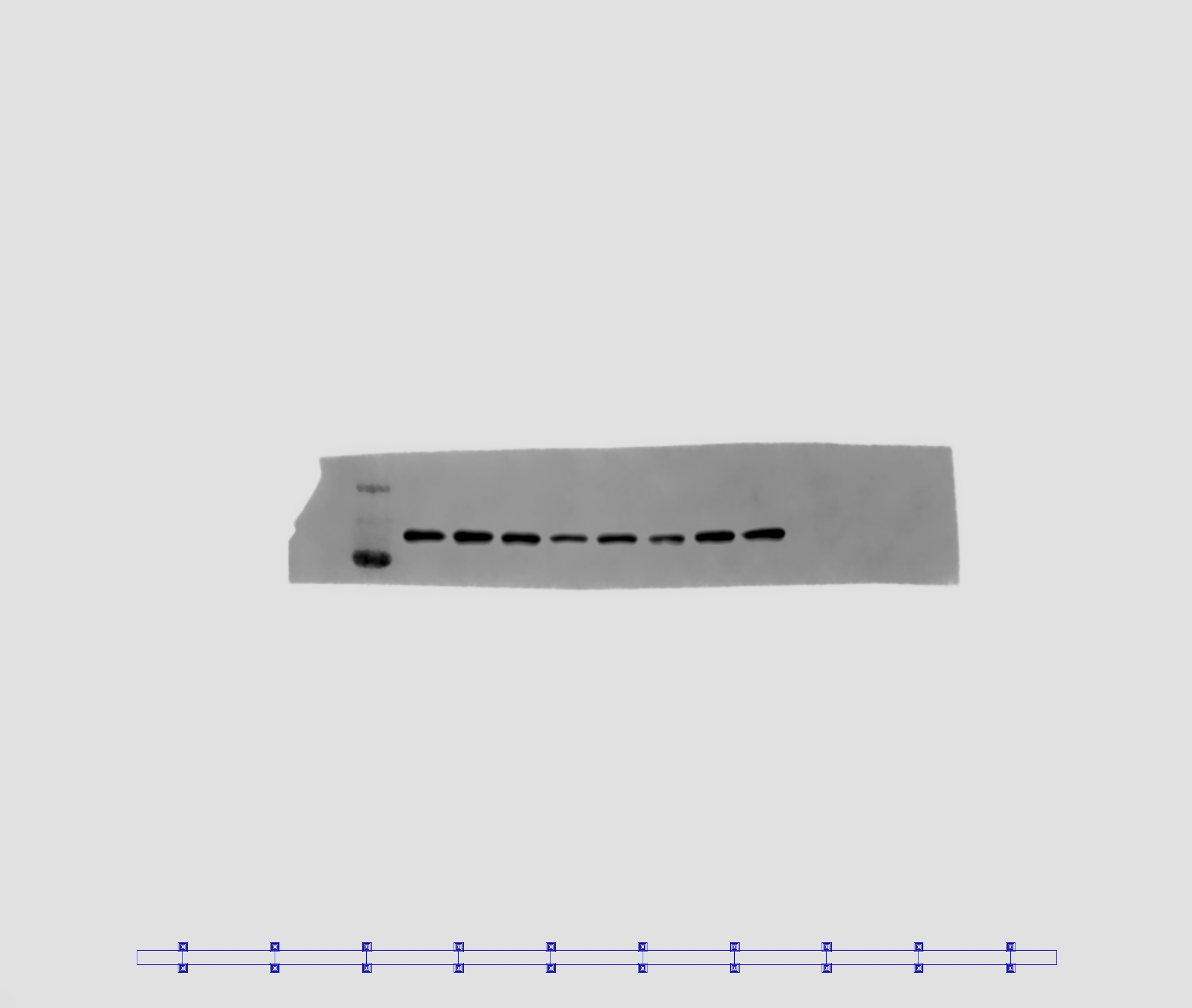

Supplement: Supplementary file 9 — Source data [file 41467_2023_43526_MOESM9_ESM.zip › Source Data/WB and Co-IP replications and quantification/Figuer.6c/replication_1/P-Rb.jpg]

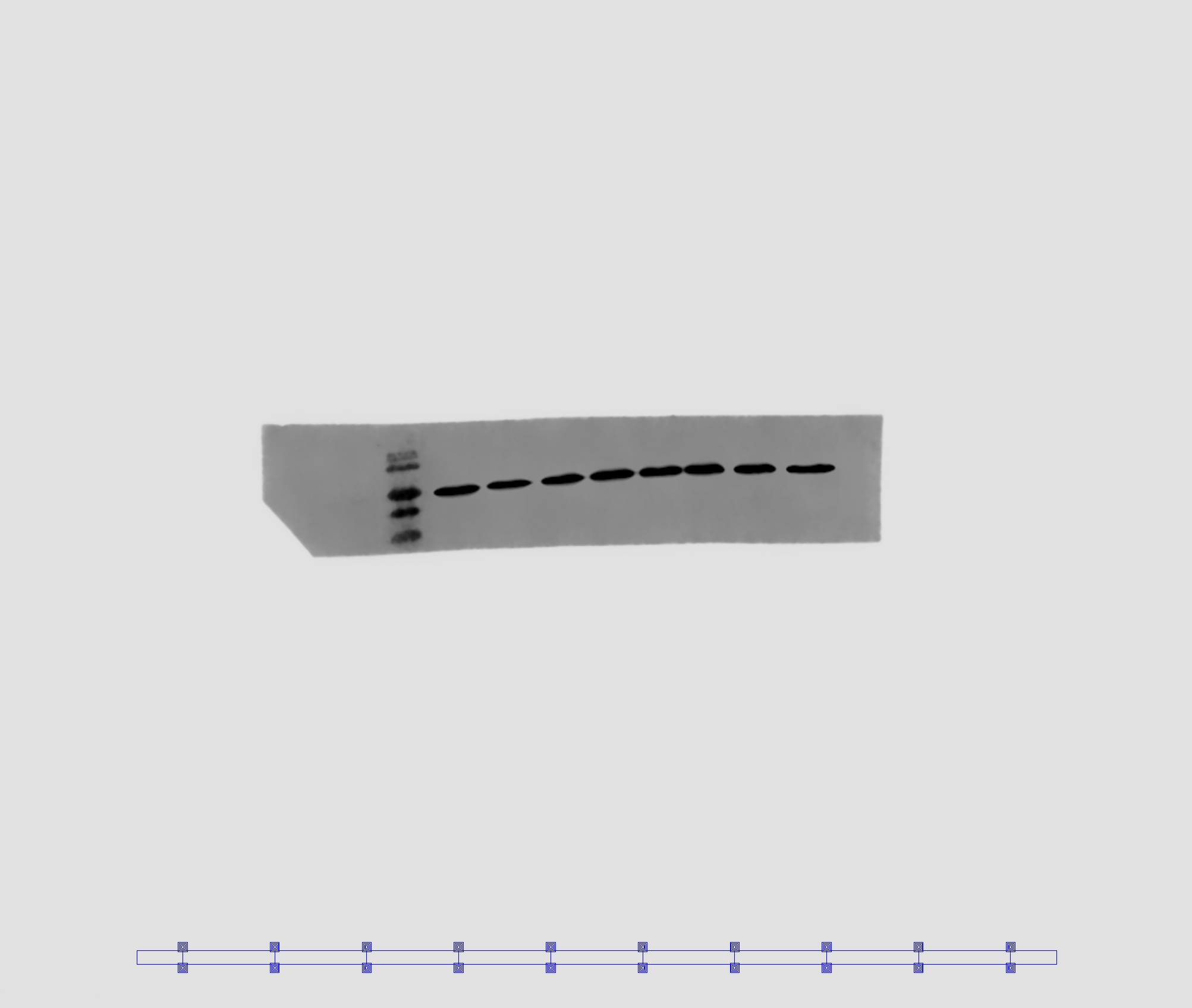

Supplement: Supplementary file 9 — Source data [file 41467_2023_43526_MOESM9_ESM.zip › Source Data/WB and Co-IP replications and quantification/Figuer.6c/replication_1/Tublin(P-H3).jpg]

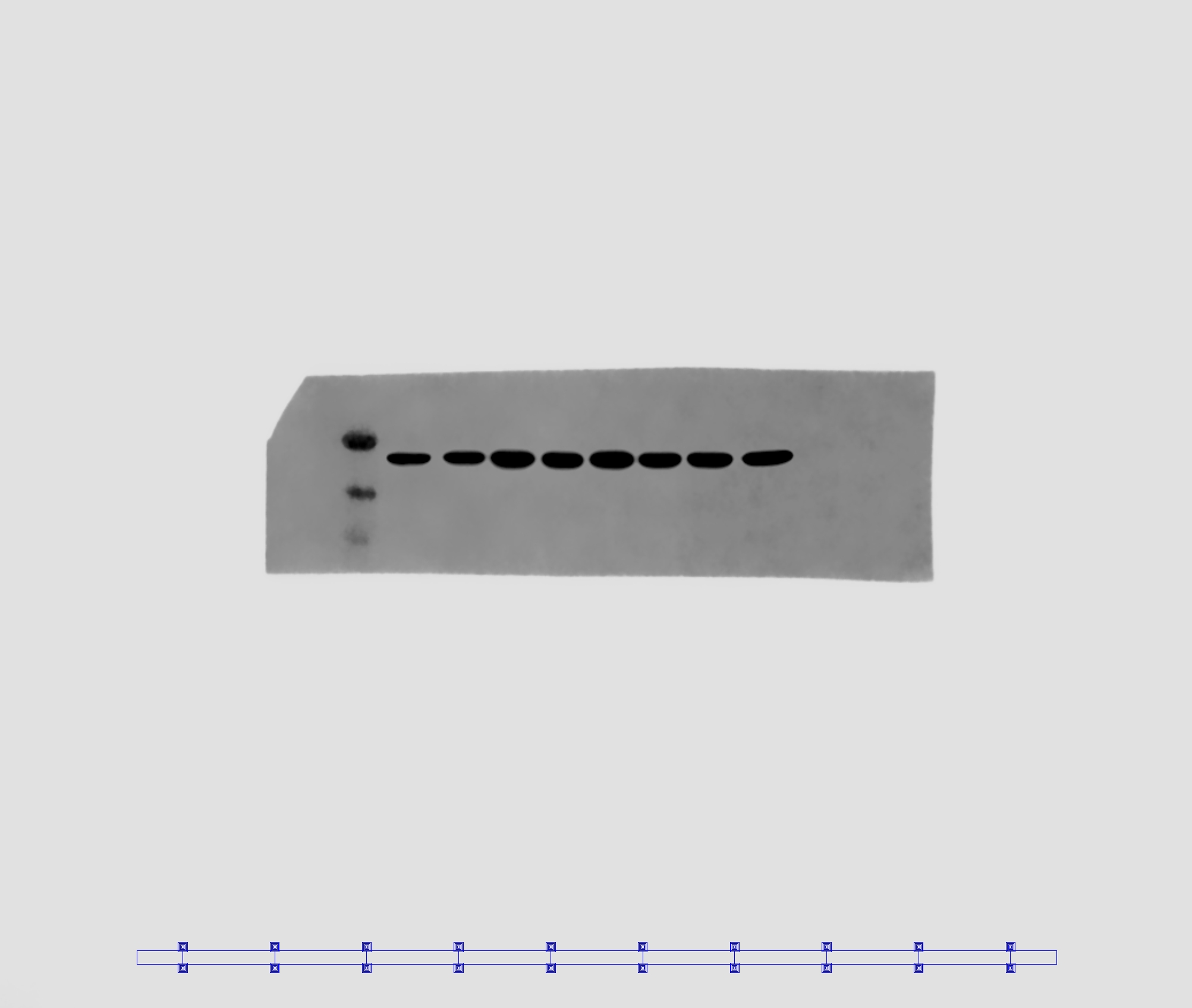

Supplement: Supplementary file 9 — Source data [file 41467_2023_43526_MOESM9_ESM.zip › Source Data/WB and Co-IP replications and quantification/Figuer.6c/replication_1/Tublin(P-Rb).jpg]

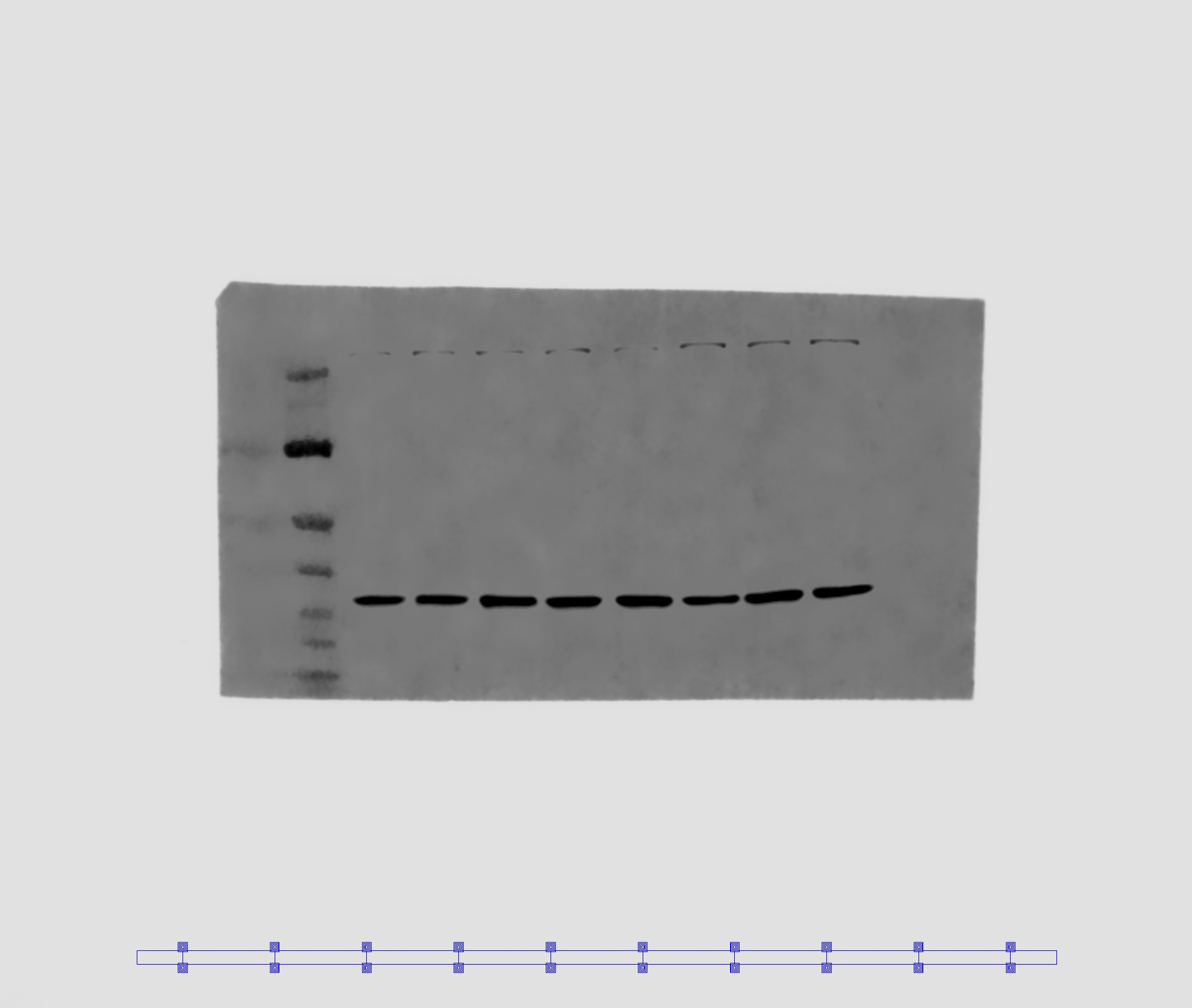

Supplement: Supplementary file 9 — Source data [file 41467_2023_43526_MOESM9_ESM.zip › Source Data/WB and Co-IP replications and quantification/Figuer.6c/replication_2/GAPDH.jpg]

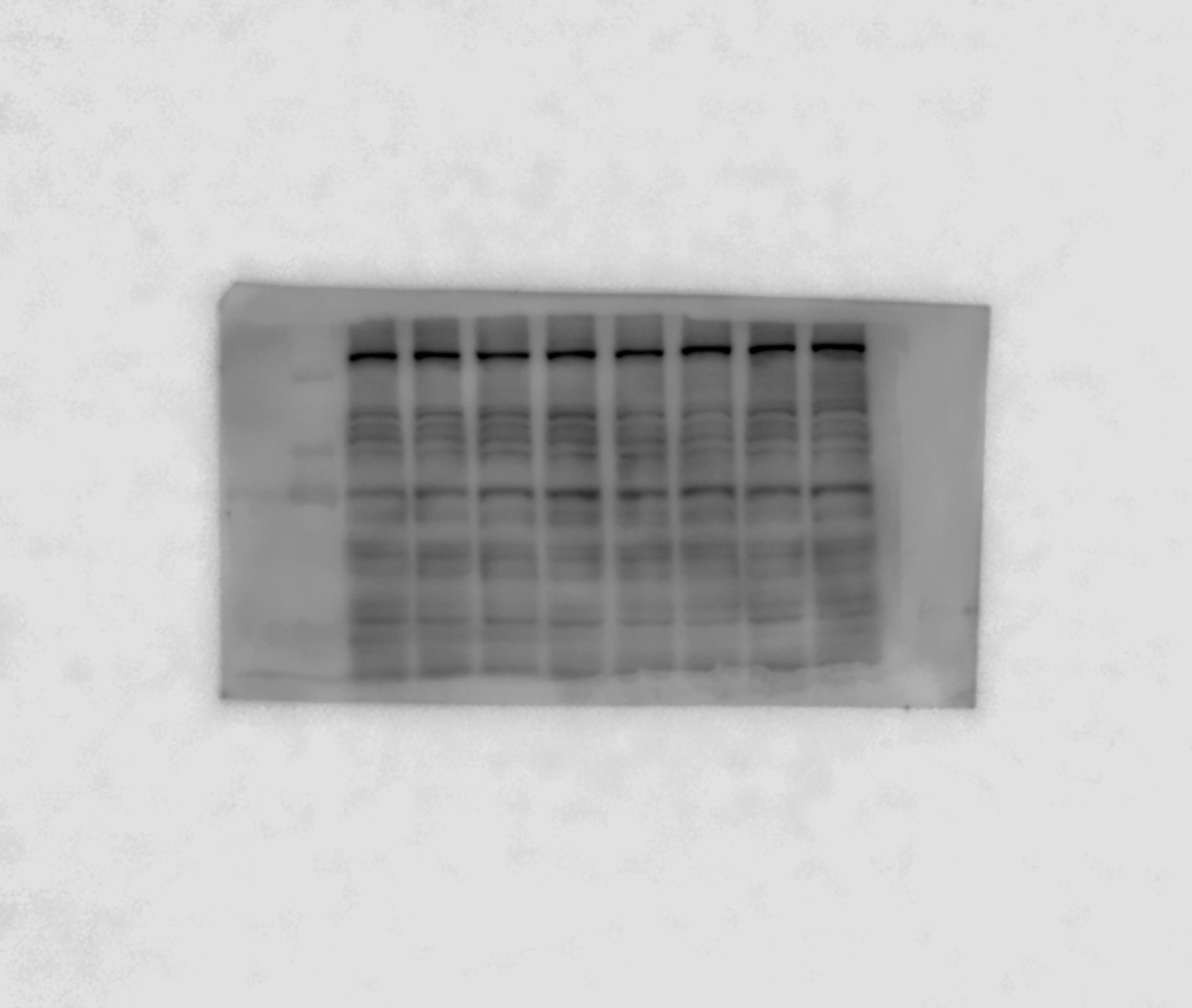

Supplement: Supplementary file 9 — Source data [file 41467_2023_43526_MOESM9_ESM.zip › Source Data/WB and Co-IP replications and quantification/Figuer.6c/replication_2/P-CDK.jpg]

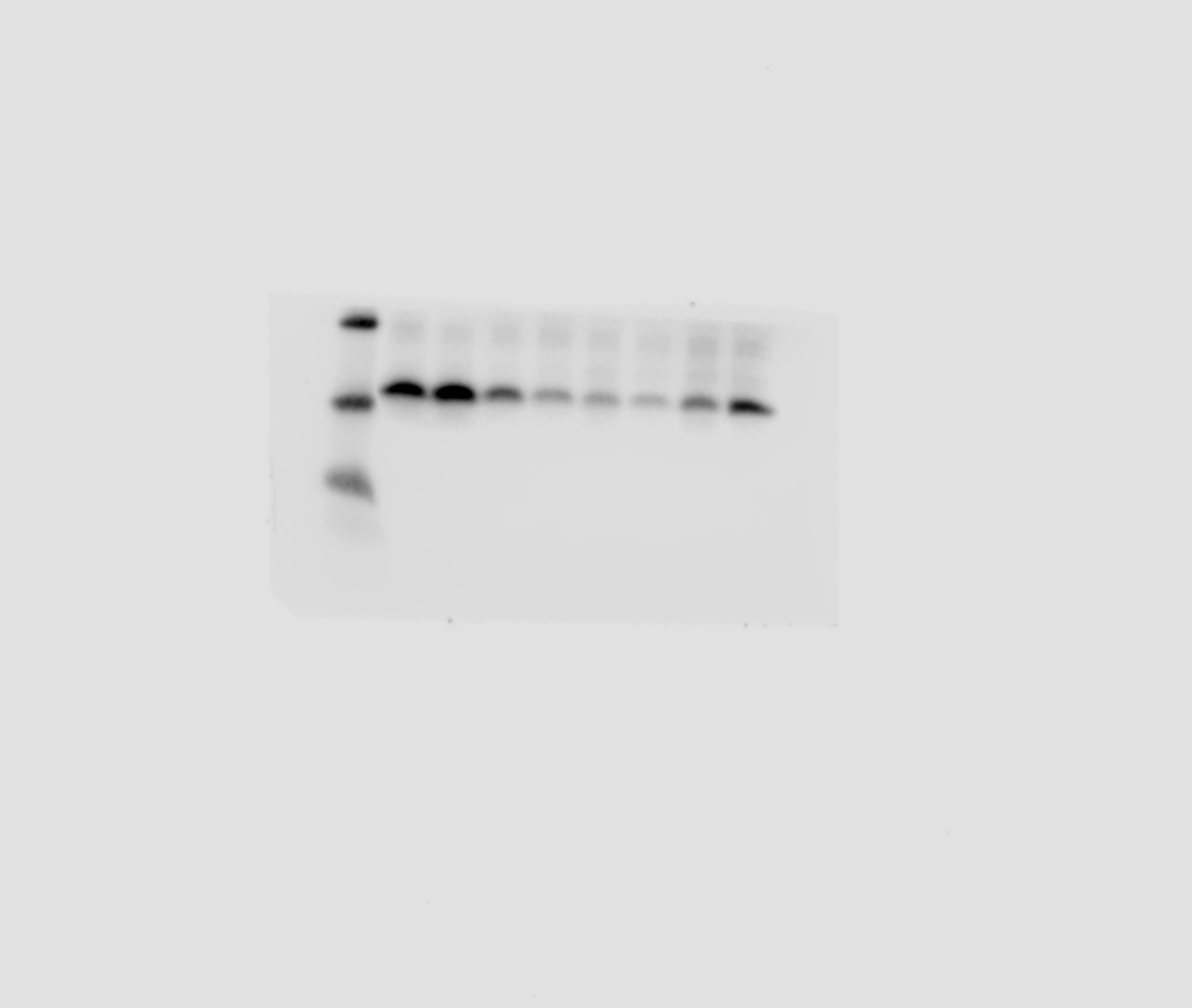

Supplement: Supplementary file 9 — Source data [file 41467_2023_43526_MOESM9_ESM.zip › Source Data/WB and Co-IP replications and quantification/Figuer.6c/replication_2/P-H3.jpg]

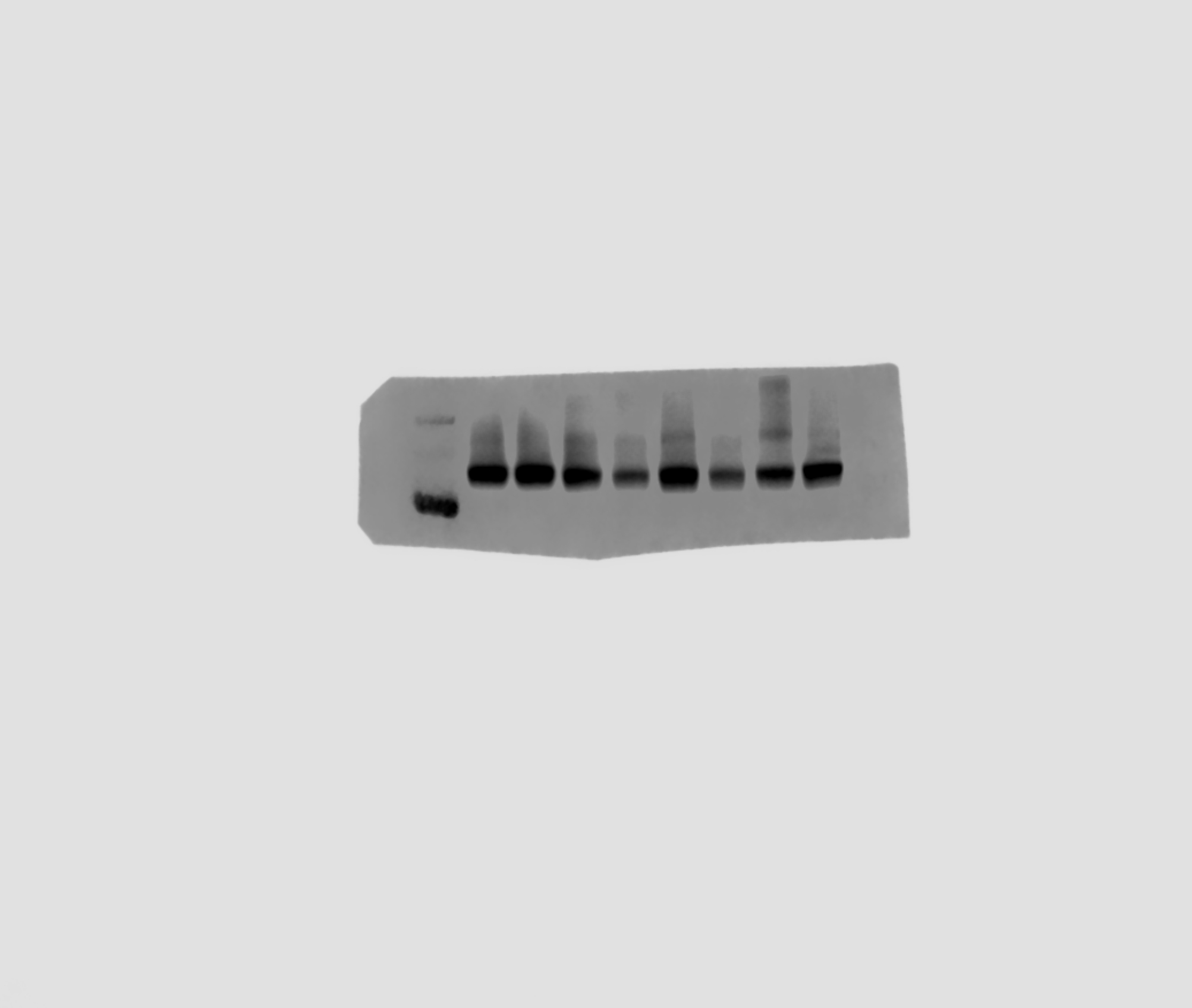

Supplement: Supplementary file 9 — Source data [file 41467_2023_43526_MOESM9_ESM.zip › Source Data/WB and Co-IP replications and quantification/Figuer.6c/replication_2/P-Rb.jpg]

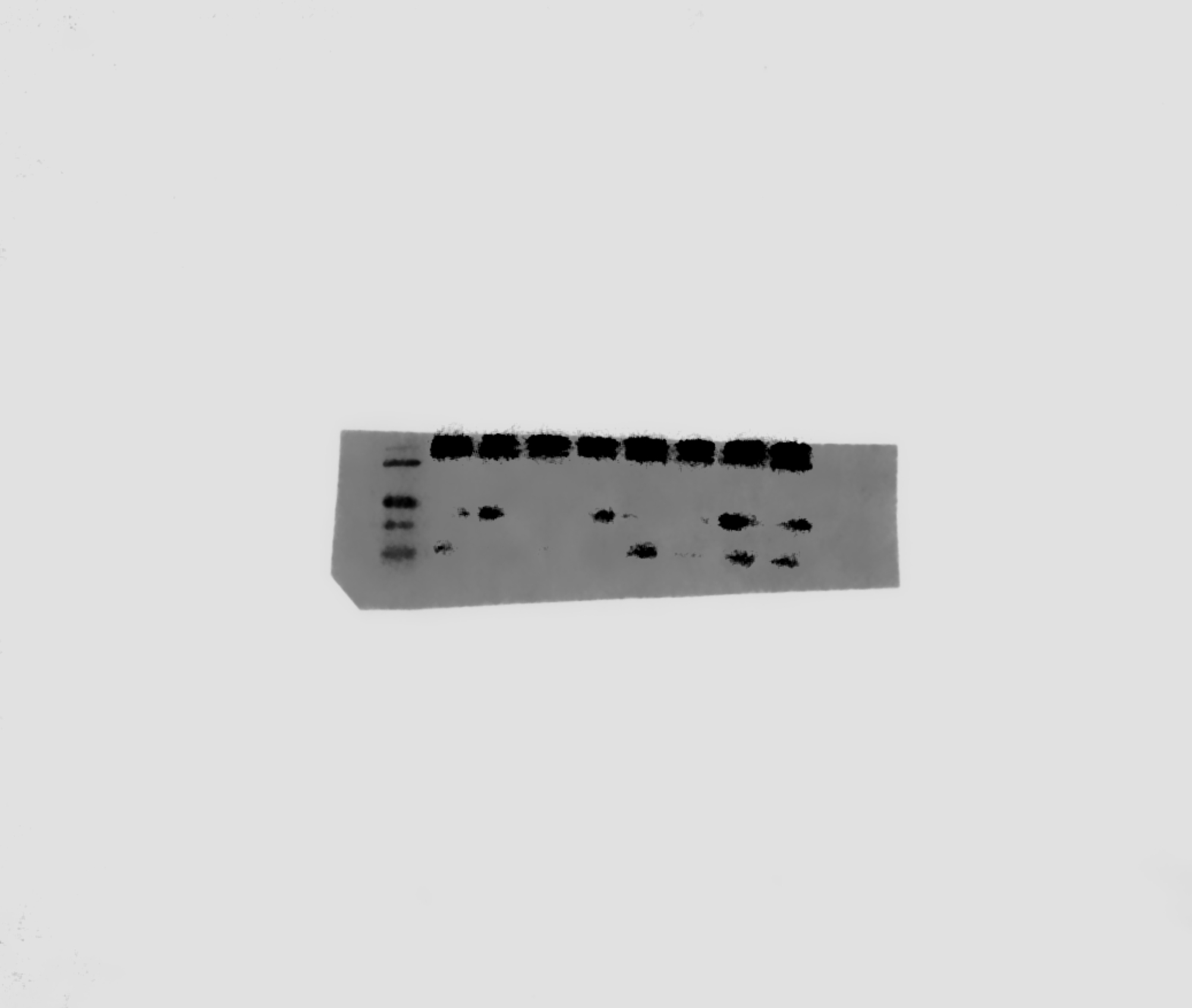

Supplement: Supplementary file 9 — Source data [file 41467_2023_43526_MOESM9_ESM.zip › Source Data/WB and Co-IP replications and quantification/Figuer.6c/replication_2/Tublin(P-H3).jpg]

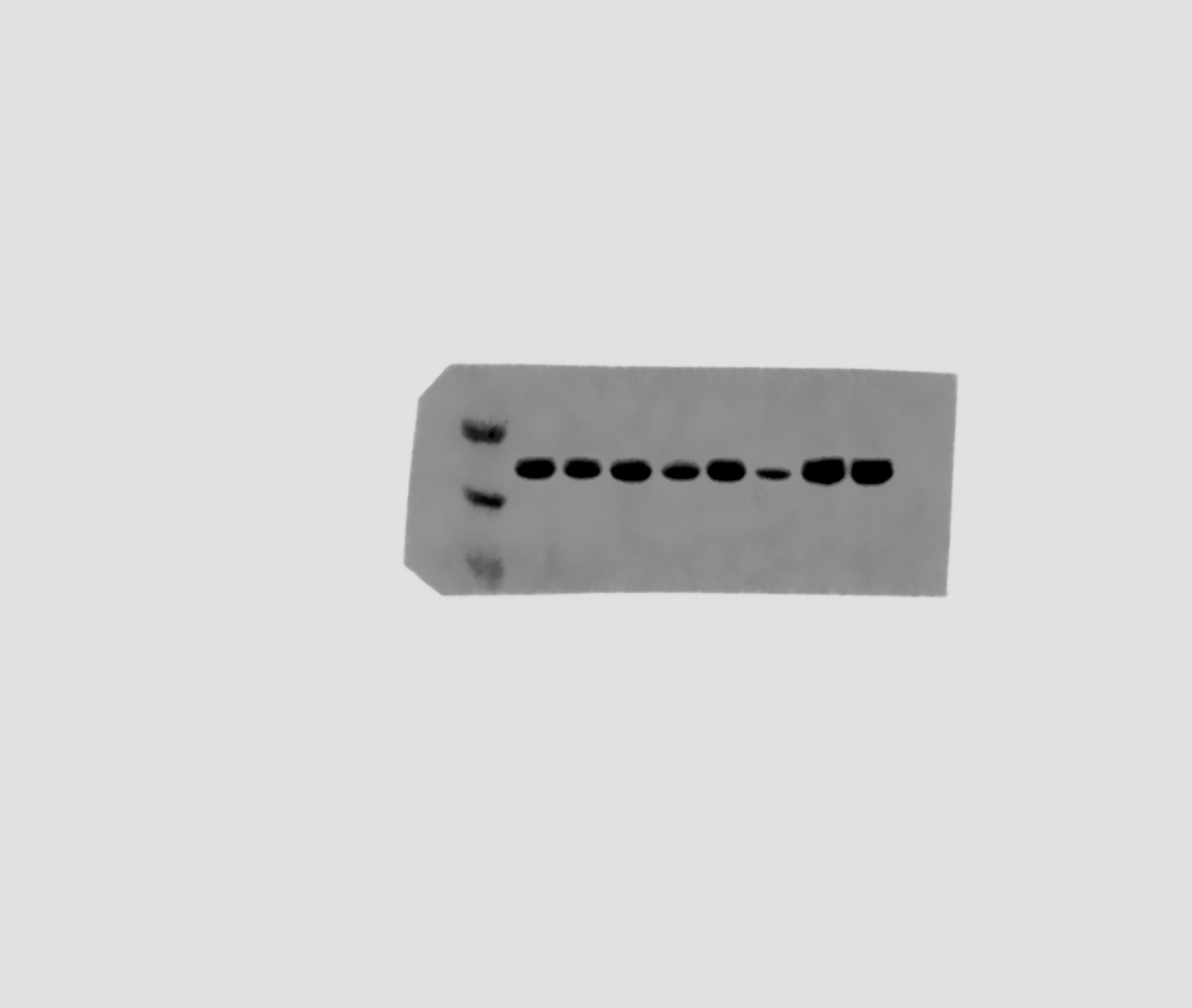

Supplement: Supplementary file 9 — Source data [file 41467_2023_43526_MOESM9_ESM.zip › Source Data/WB and Co-IP replications and quantification/Figuer.6c/replication_2/Tublin(P-Rb).tif]

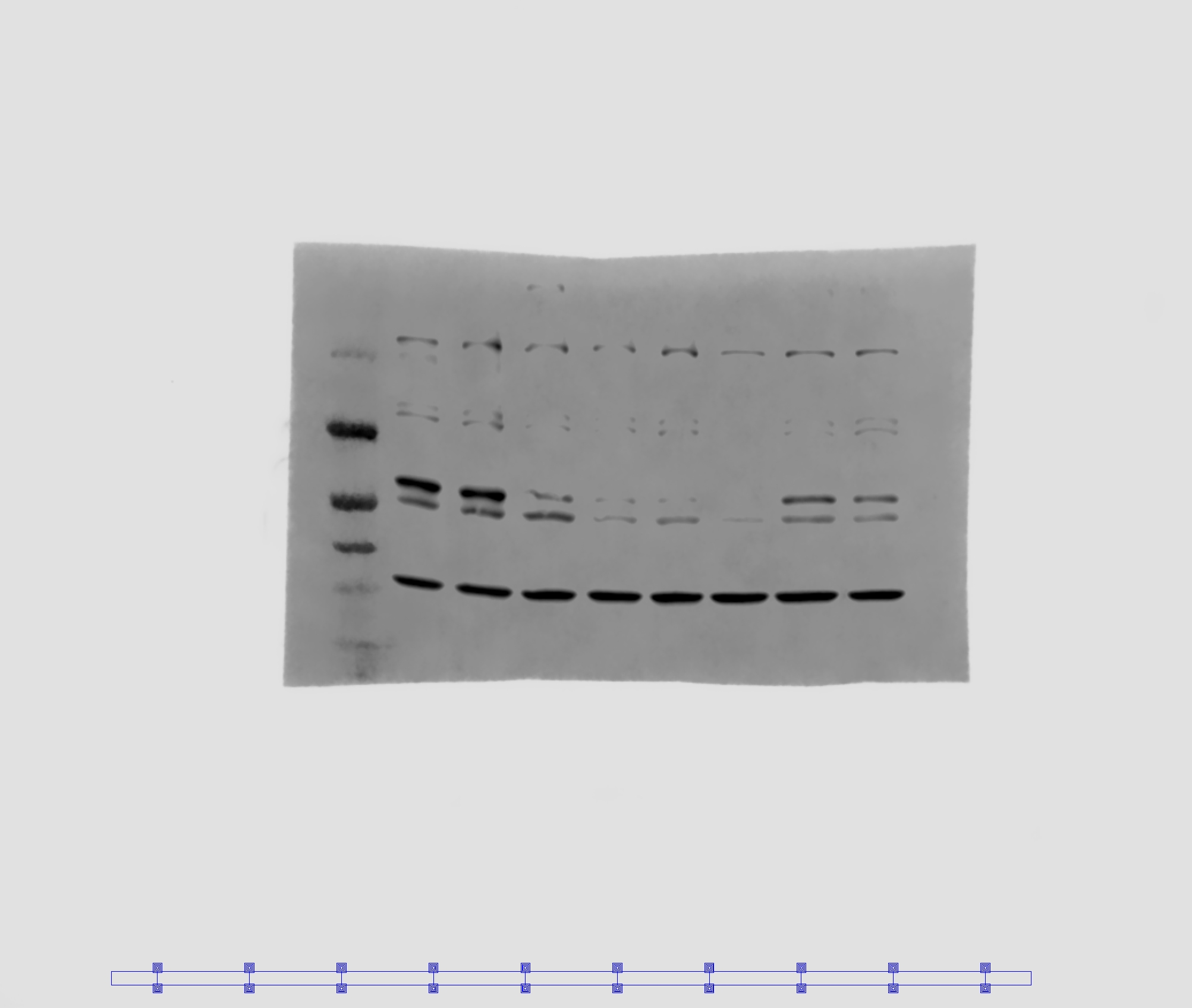

Supplement: Supplementary file 9 — Source data [file 41467_2023_43526_MOESM9_ESM.zip › Source Data/WB and Co-IP replications and quantification/Figuer.6c/replication_3/GAPDH.jpg]

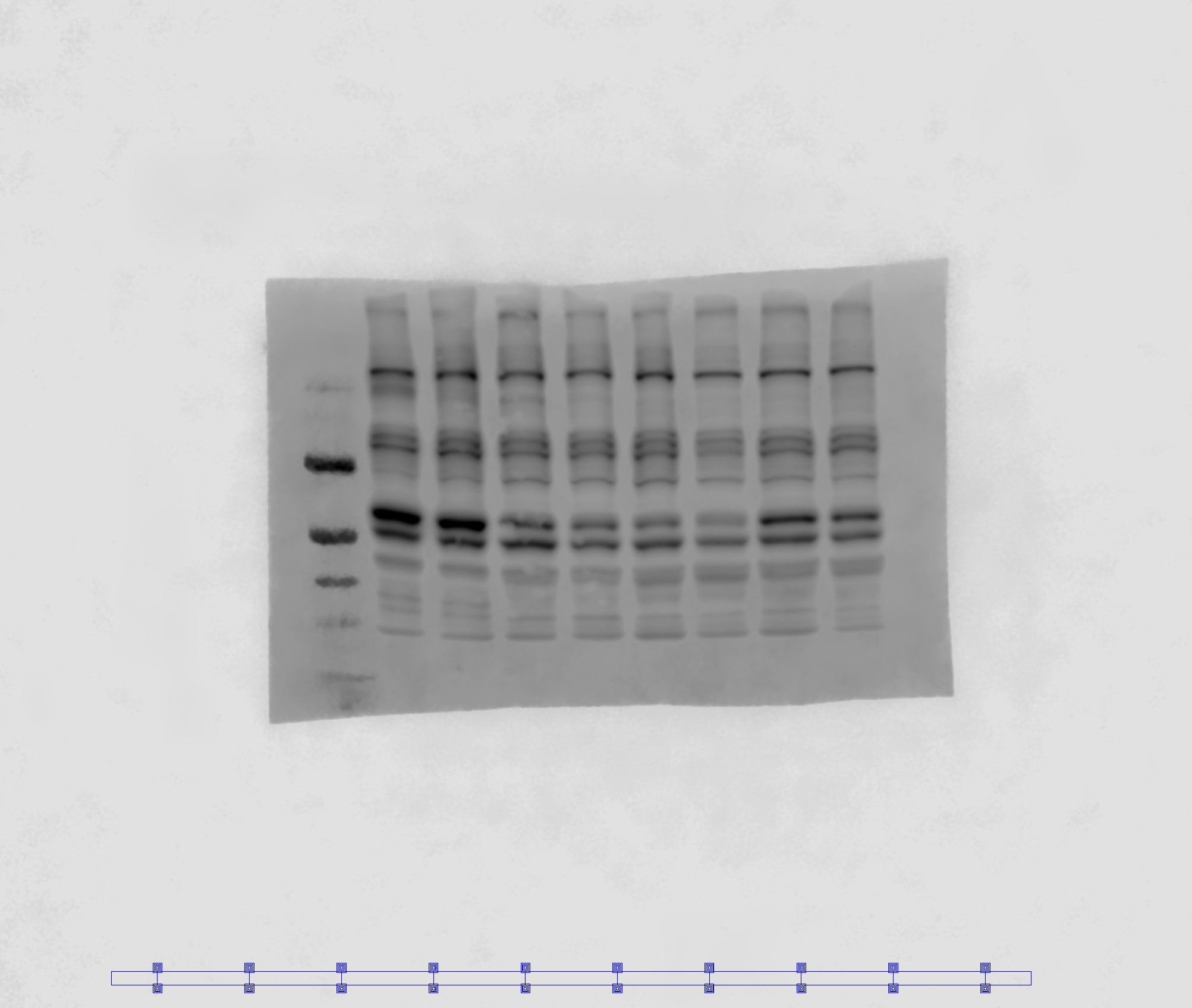

Supplement: Supplementary file 9 — Source data [file 41467_2023_43526_MOESM9_ESM.zip › Source Data/WB and Co-IP replications and quantification/Figuer.6c/replication_3/P-CDK.tif]

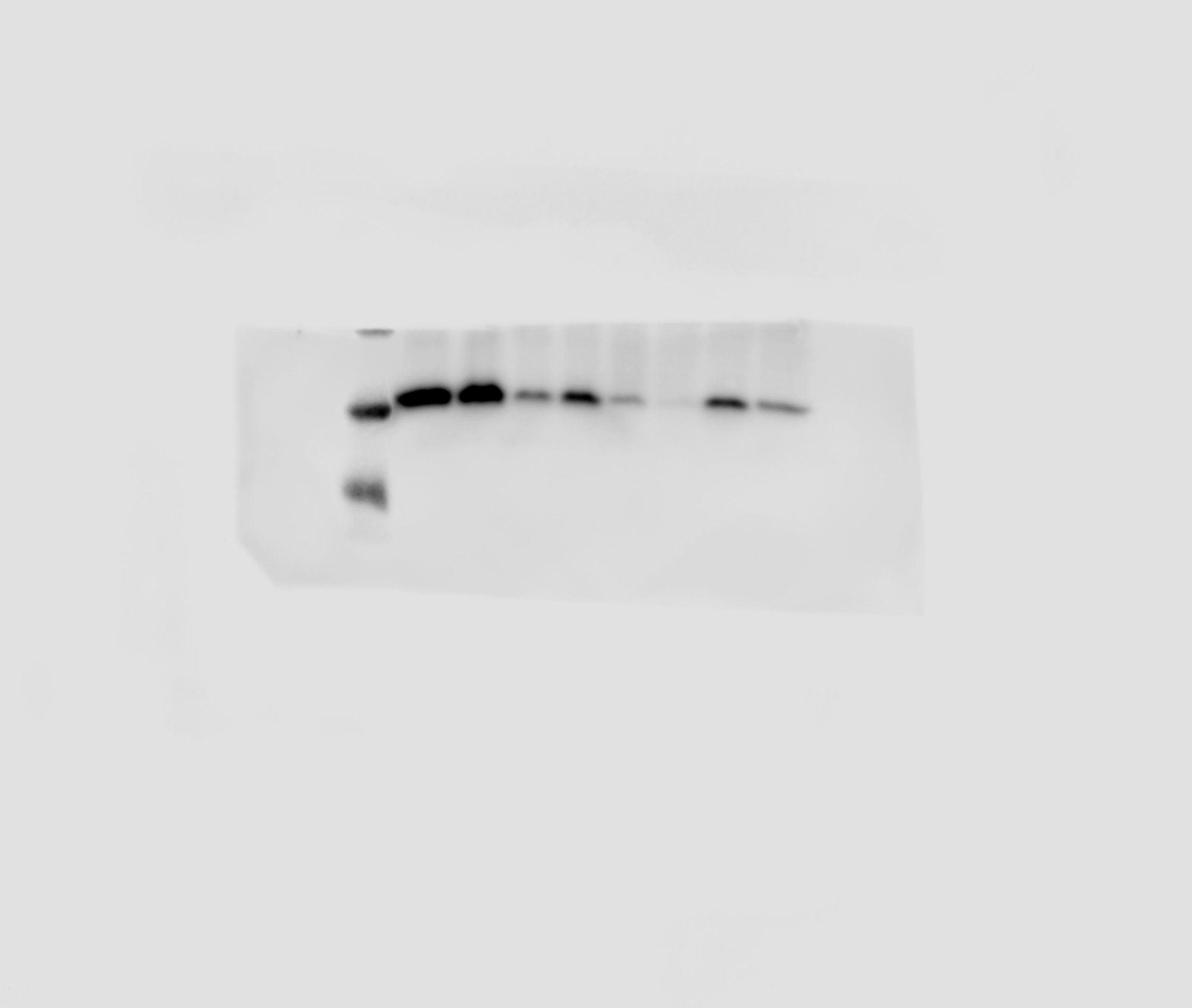

Supplement: Supplementary file 9 — Source data [file 41467_2023_43526_MOESM9_ESM.zip › Source Data/WB and Co-IP replications and quantification/Figuer.6c/replication_3/P-H3.jpg]

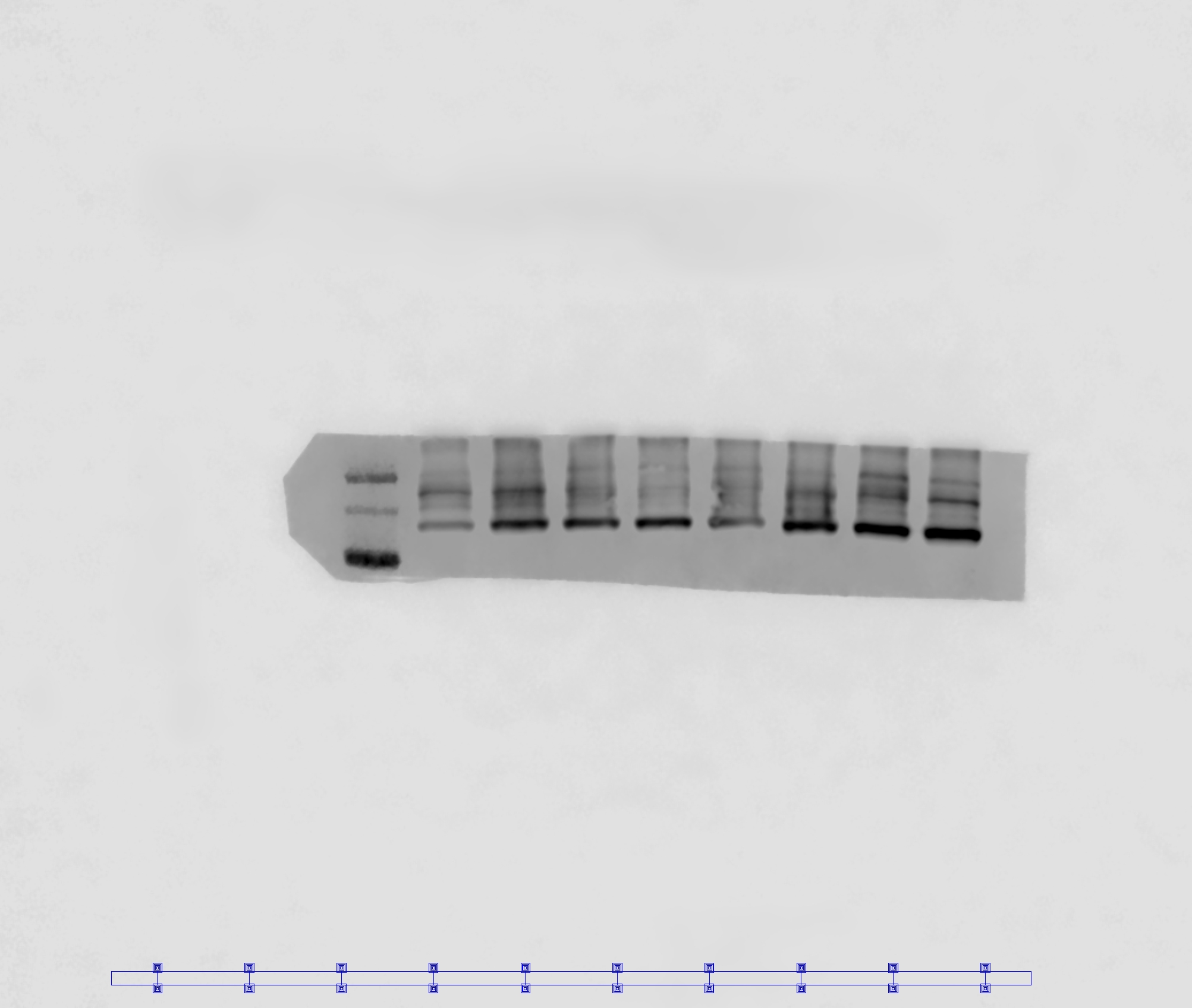

Supplement: Supplementary file 9 — Source data [file 41467_2023_43526_MOESM9_ESM.zip › Source Data/WB and Co-IP replications and quantification/Figuer.6c/replication_3/P-Rb.jpg]

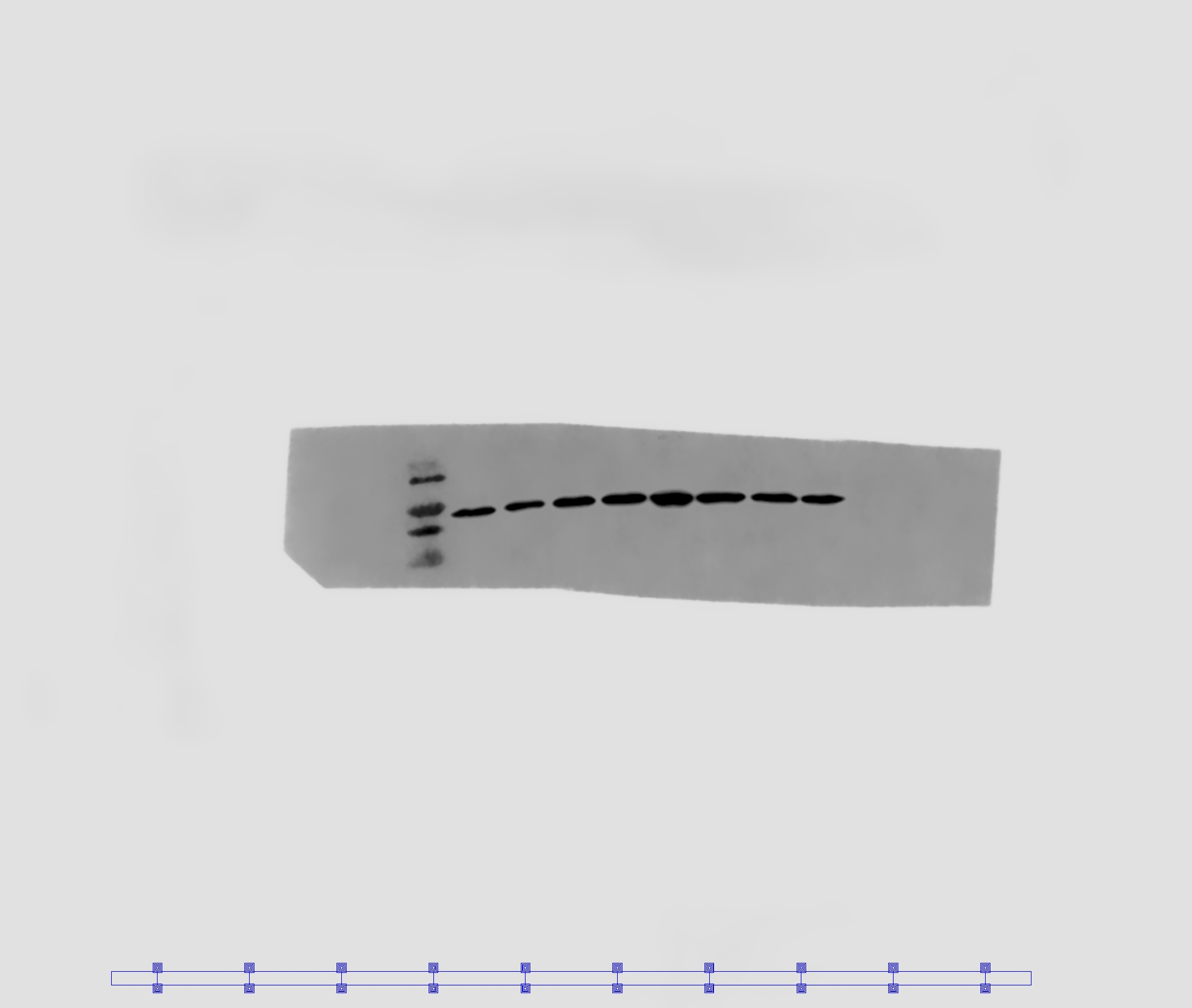

Supplement: Supplementary file 9 — Source data [file 41467_2023_43526_MOESM9_ESM.zip › Source Data/WB and Co-IP replications and quantification/Figuer.6c/replication_3/Tublin(P-H3).jpg]

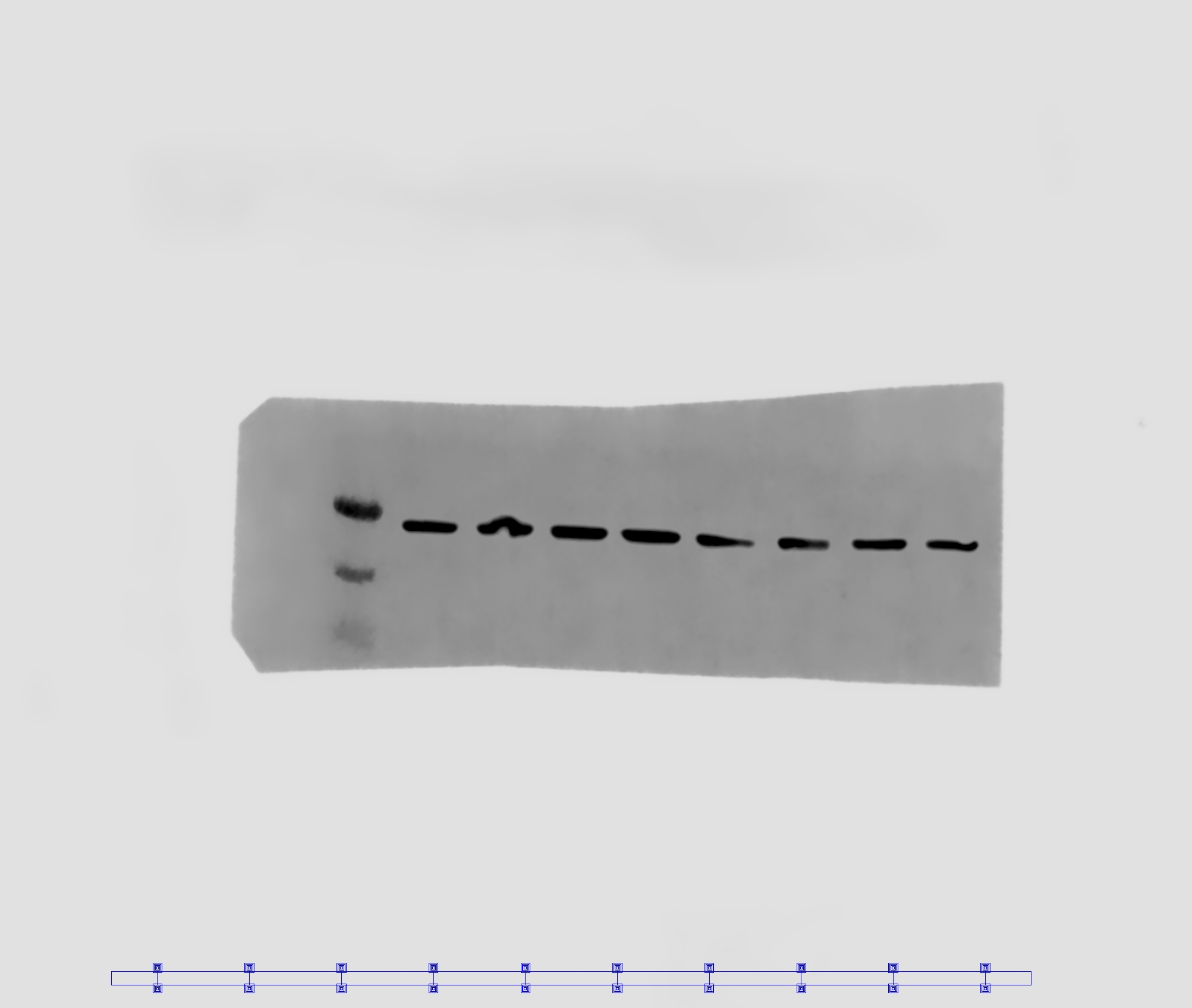

Supplement: Supplementary file 9 — Source data [file 41467_2023_43526_MOESM9_ESM.zip › Source Data/WB and Co-IP replications and quantification/Figuer.6c/replication_3/Tublin(P-Rb).jpg]

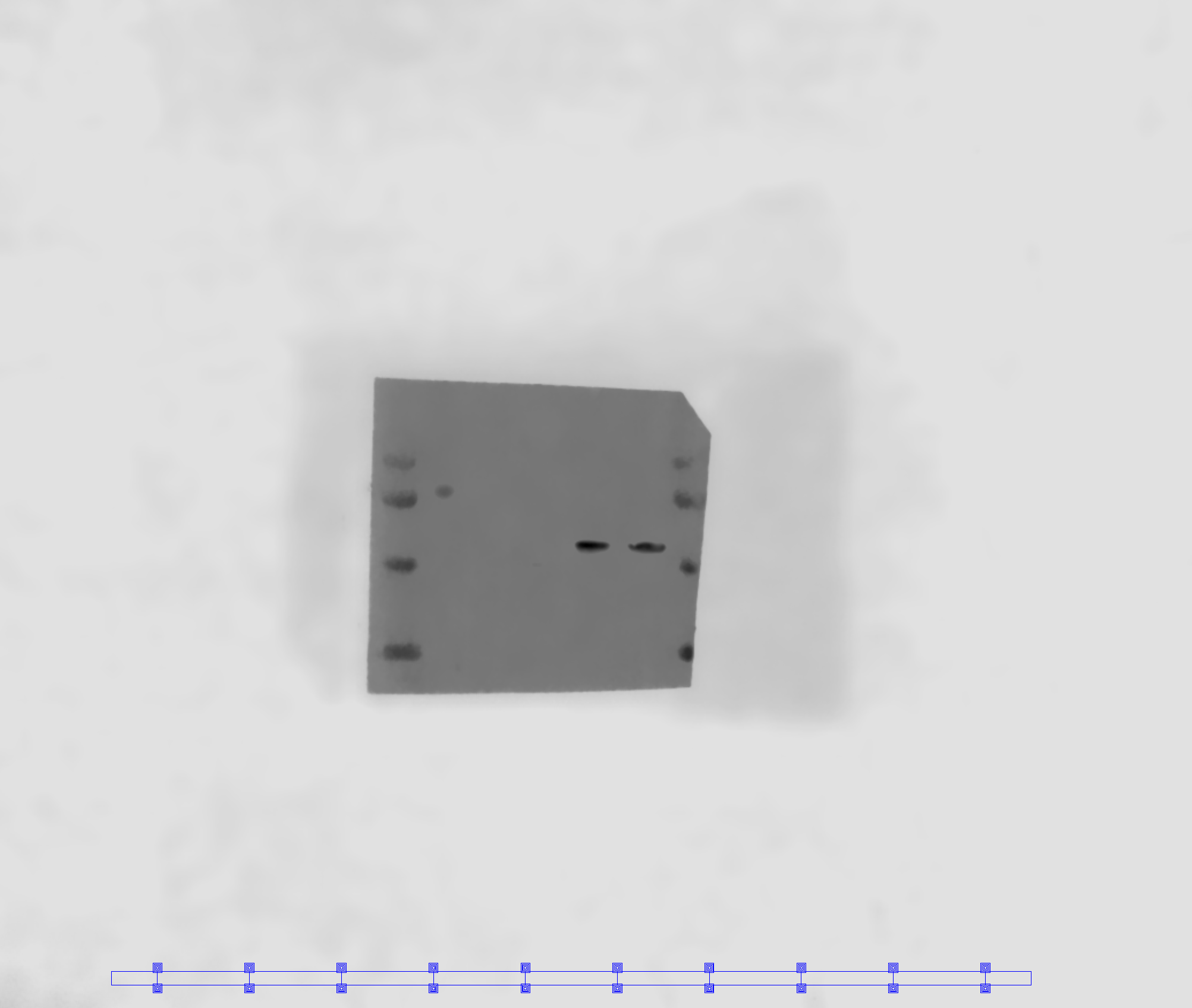

Supplement: Supplementary file 9 — Source data [file 41467_2023_43526_MOESM9_ESM.zip › Source Data/WB and Co-IP replications and quantification/Figuer.6d/replication_1/GAPDH.tif]

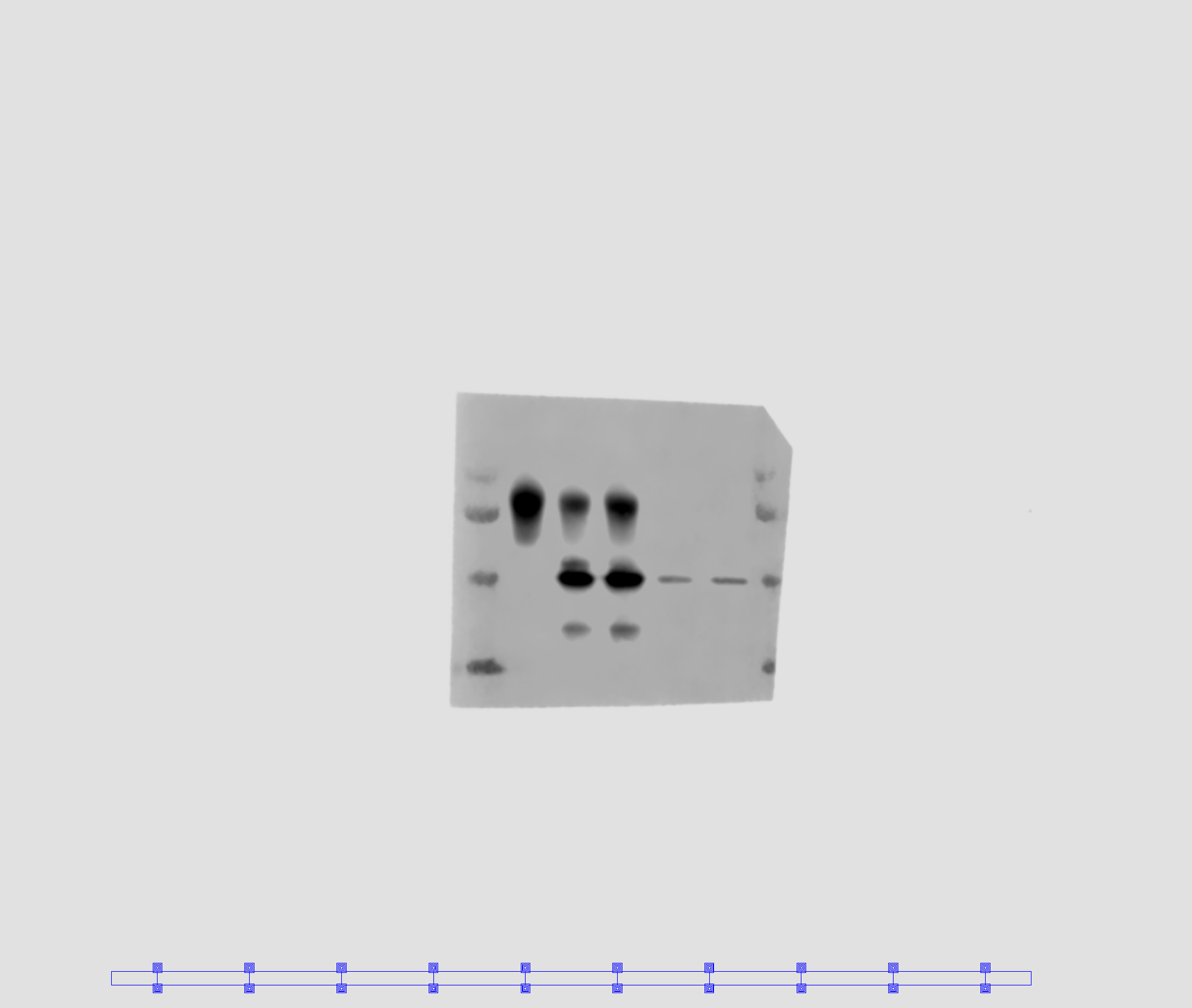

Supplement: Supplementary file 9 — Source data [file 41467_2023_43526_MOESM9_ESM.zip › Source Data/WB and Co-IP replications and quantification/Figuer.6d/replication_1/IP-Emerin.tif]

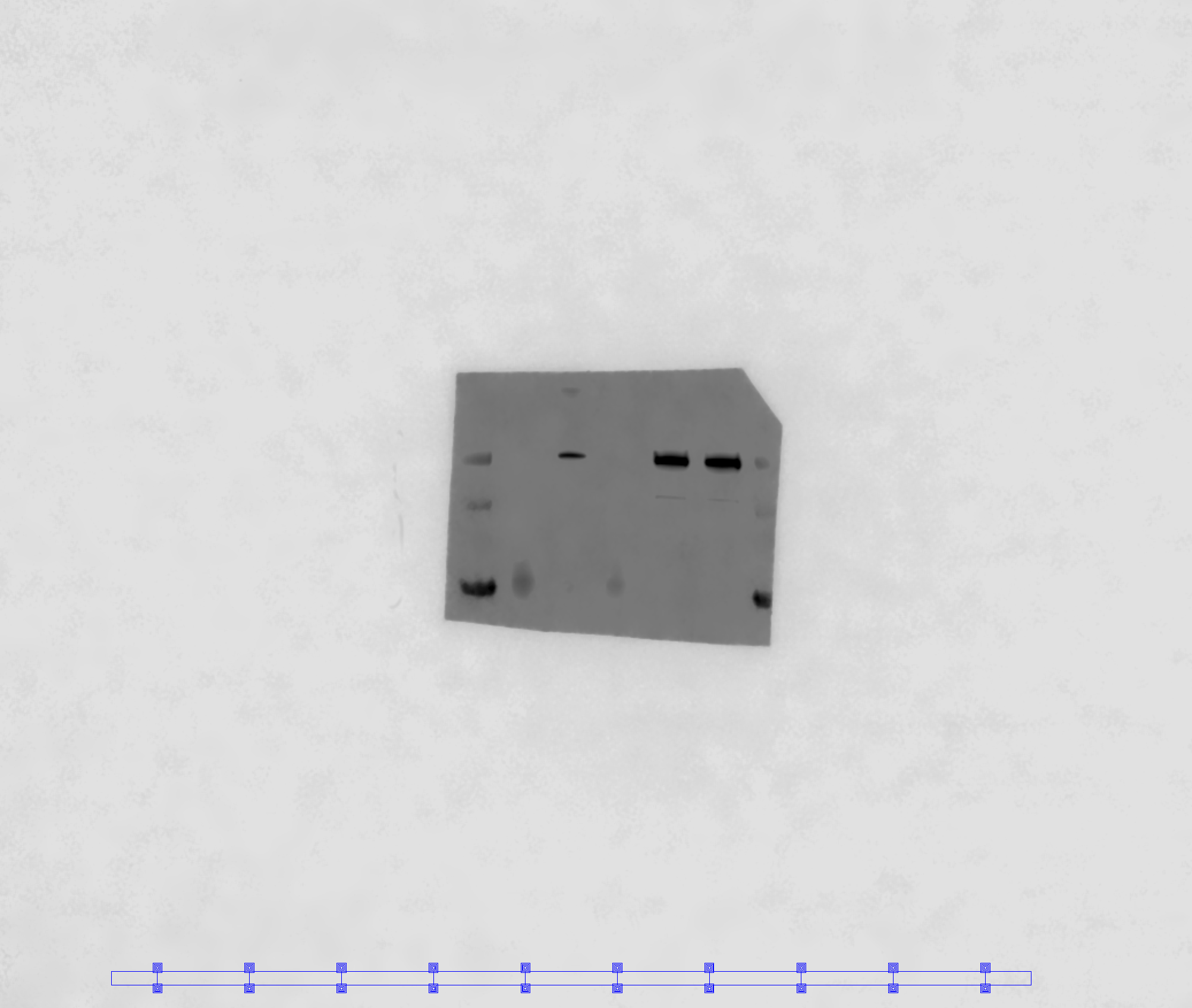

Supplement: Supplementary file 9 — Source data [file 41467_2023_43526_MOESM9_ESM.zip › Source Data/WB and Co-IP replications and quantification/Figuer.6d/replication_1/MYH9.tif]

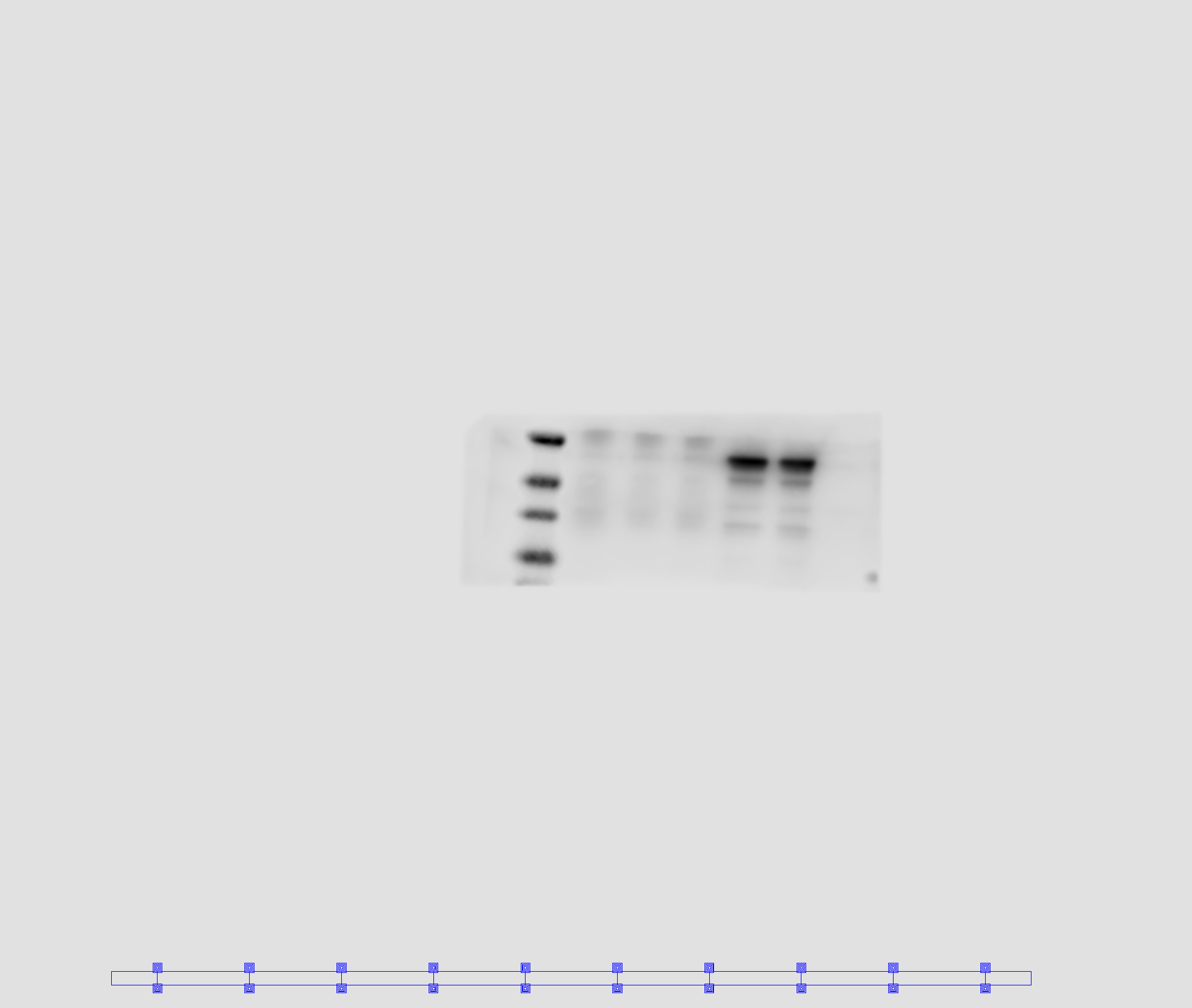

Supplement: Supplementary file 9 — Source data [file 41467_2023_43526_MOESM9_ESM.zip › Source Data/WB and Co-IP replications and quantification/Figuer.6d/replication_2/GAPDH.tif]

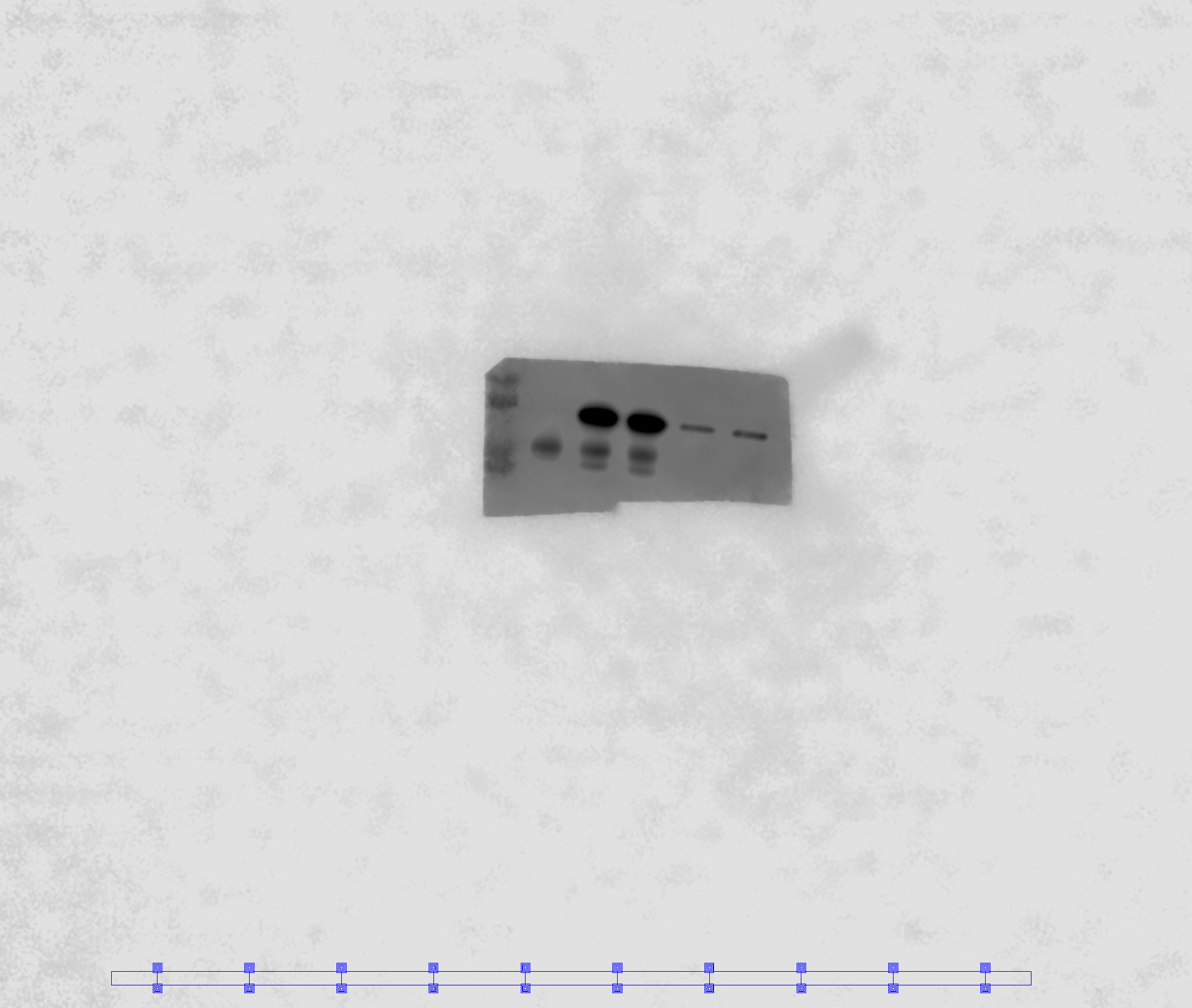

Supplement: Supplementary file 9 — Source data [file 41467_2023_43526_MOESM9_ESM.zip › Source Data/WB and Co-IP replications and quantification/Figuer.6d/replication_2/IP-Emerin.tif]

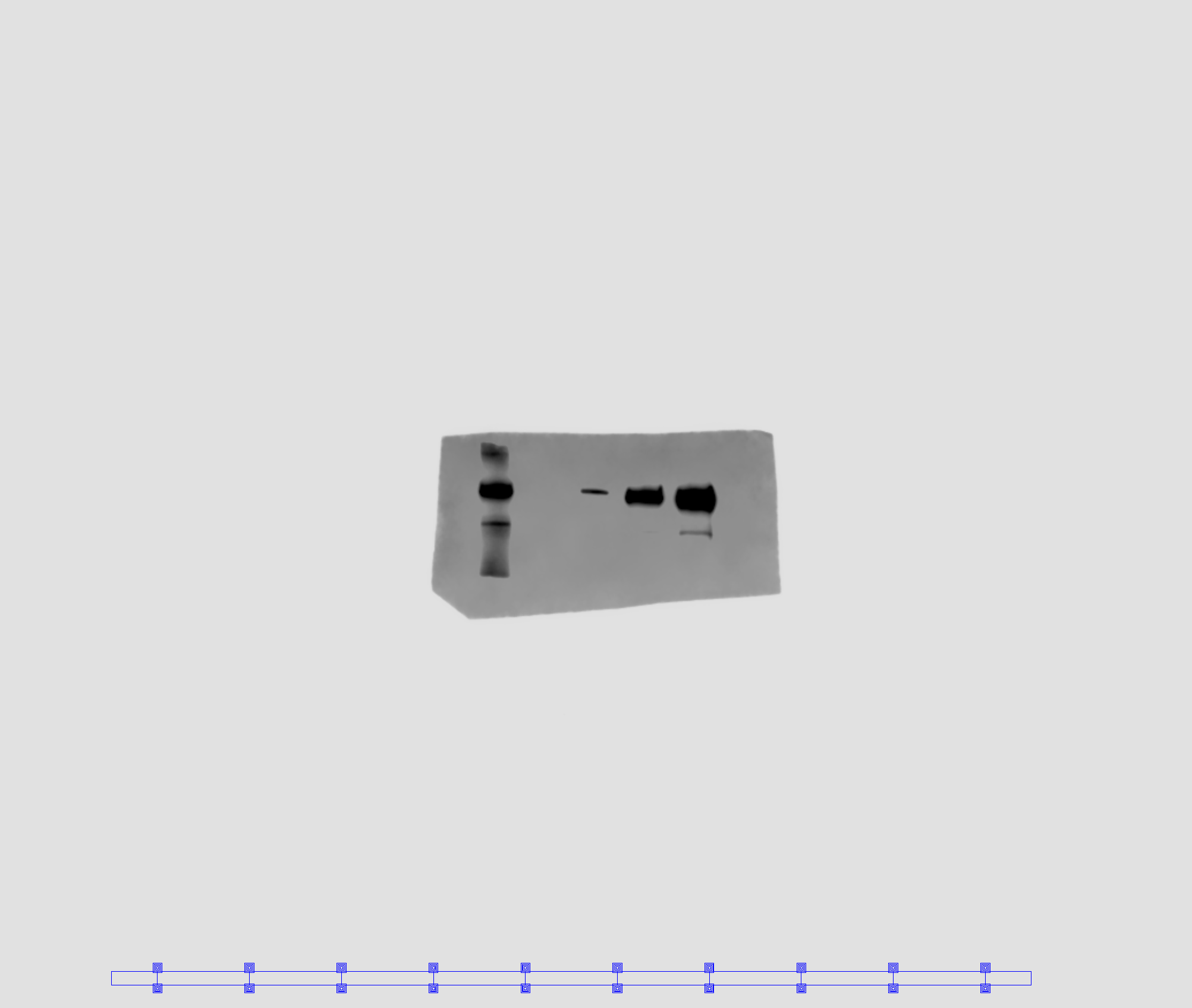

Supplement: Supplementary file 9 — Source data [file 41467_2023_43526_MOESM9_ESM.zip › Source Data/WB and Co-IP replications and quantification/Figuer.6d/replication_2/MYH9.tif]

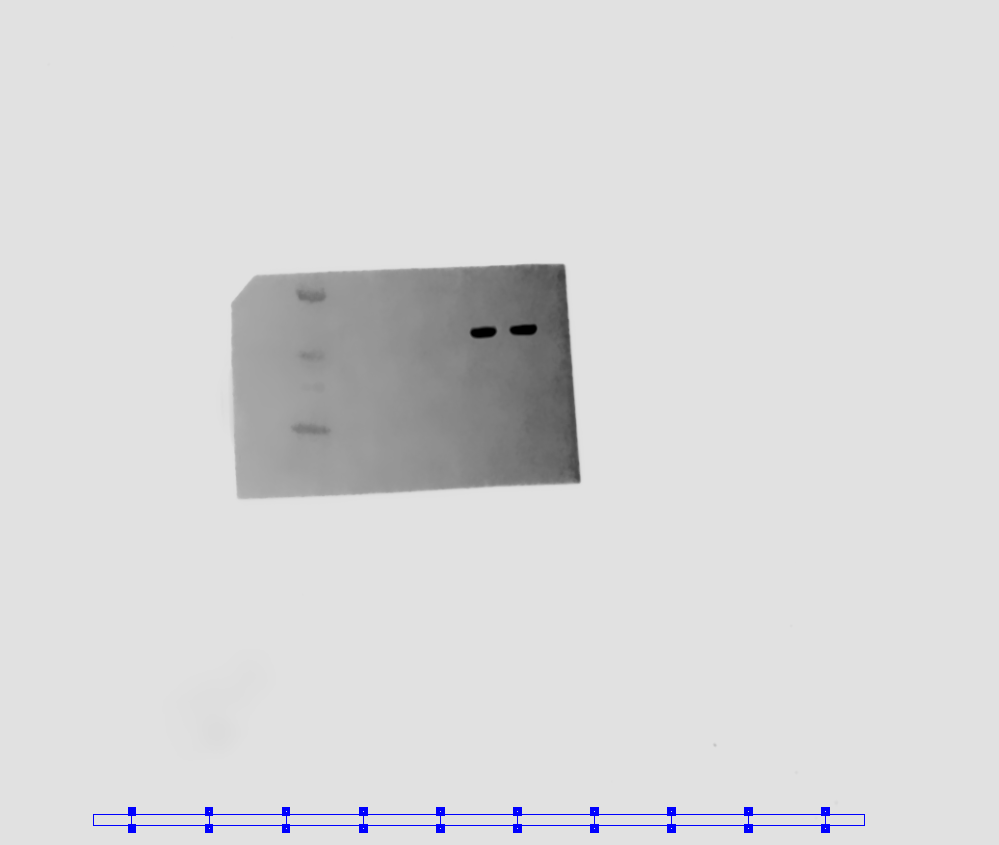

Supplement: Supplementary file 9 — Source data [file 41467_2023_43526_MOESM9_ESM.zip › Source Data/WB and Co-IP replications and quantification/Figuer.6d/replication_3/GAPDH.png]

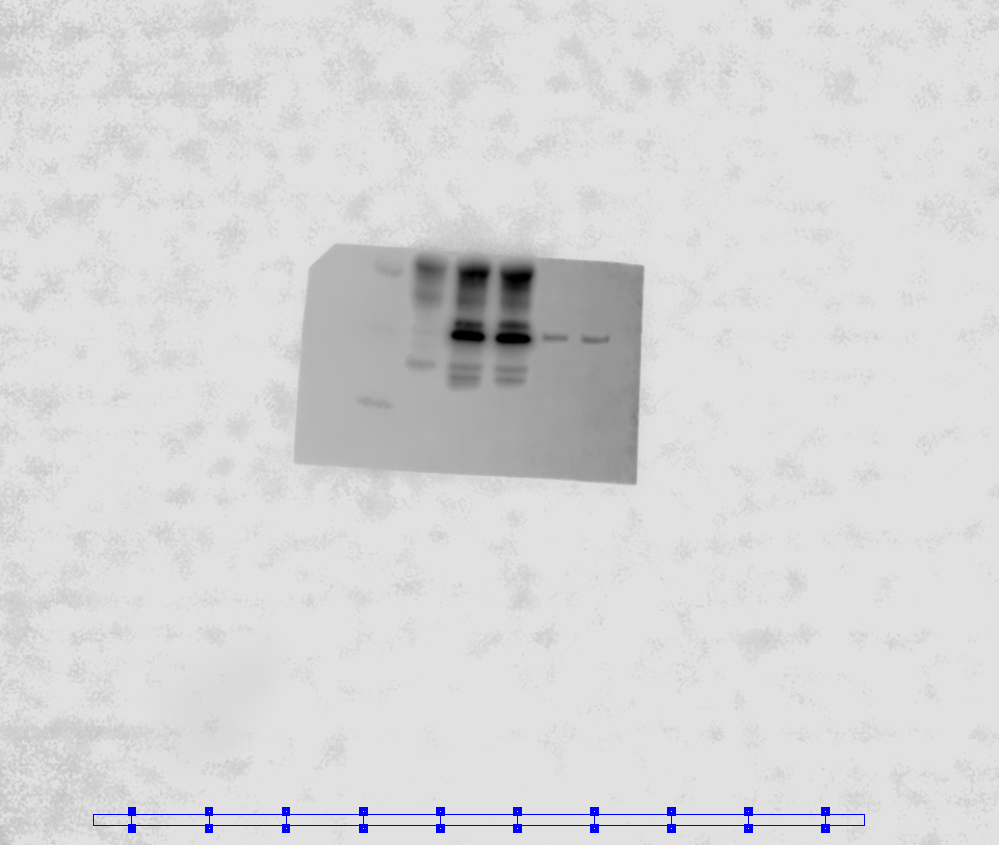

Supplement: Supplementary file 9 — Source data [file 41467_2023_43526_MOESM9_ESM.zip › Source Data/WB and Co-IP replications and quantification/Figuer.6d/replication_3/IP-EMERIN.png]

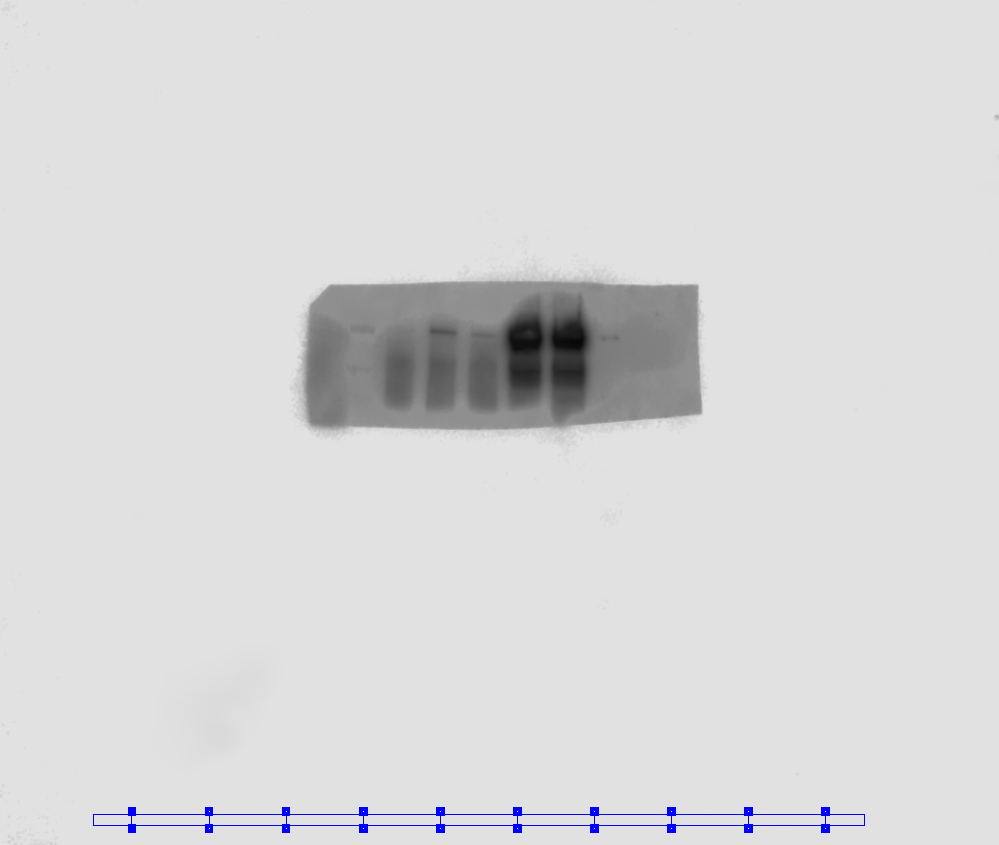

Supplement: Supplementary file 9 — Source data [file 41467_2023_43526_MOESM9_ESM.zip › Source Data/WB and Co-IP replications and quantification/Figuer.6d/replication_3/MYH9.png]

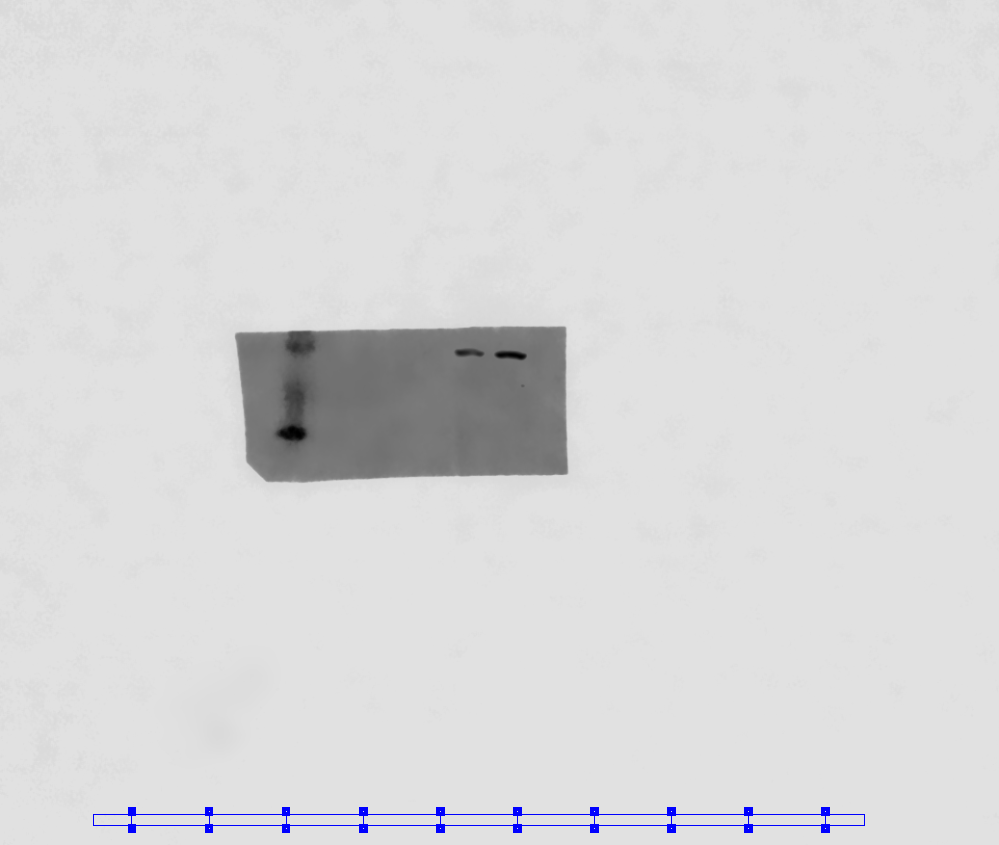

Supplement: Supplementary file 9 — Source data [file 41467_2023_43526_MOESM9_ESM.zip › Source Data/WB and Co-IP replications and quantification/Figuer.6e/replication_1/GAPDH.png]

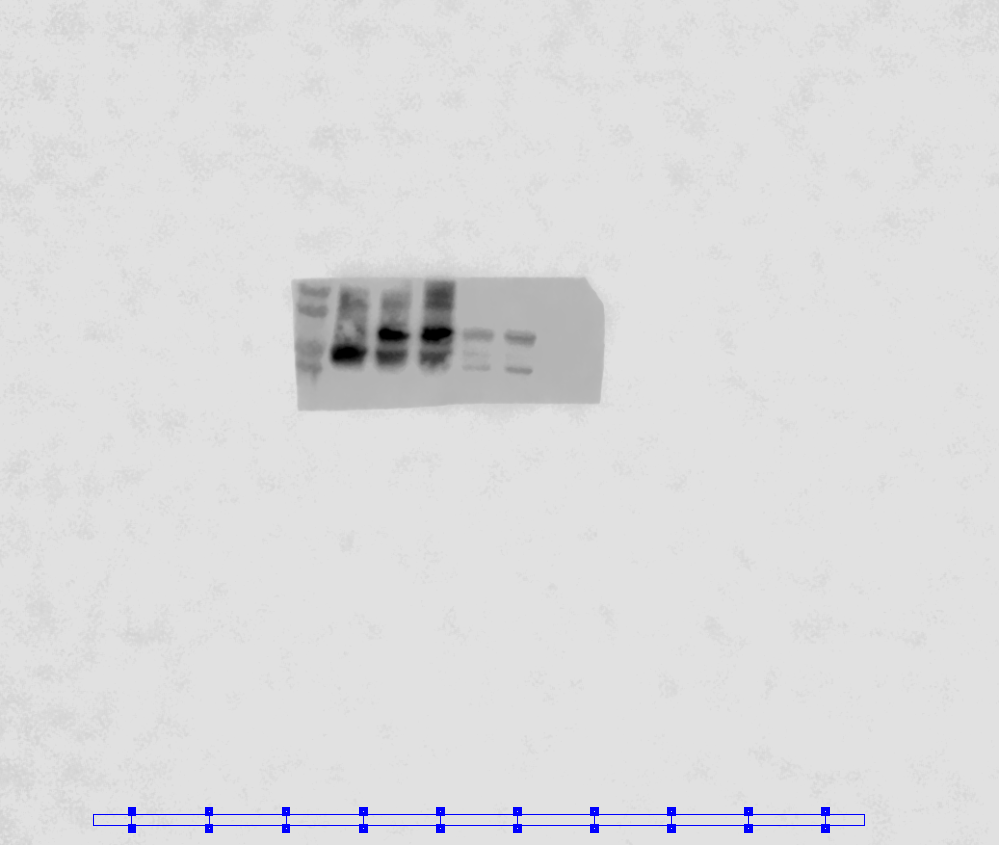

Supplement: Supplementary file 9 — Source data [file 41467_2023_43526_MOESM9_ESM.zip › Source Data/WB and Co-IP replications and quantification/Figuer.6e/replication_1/IP-TAF9B.png]

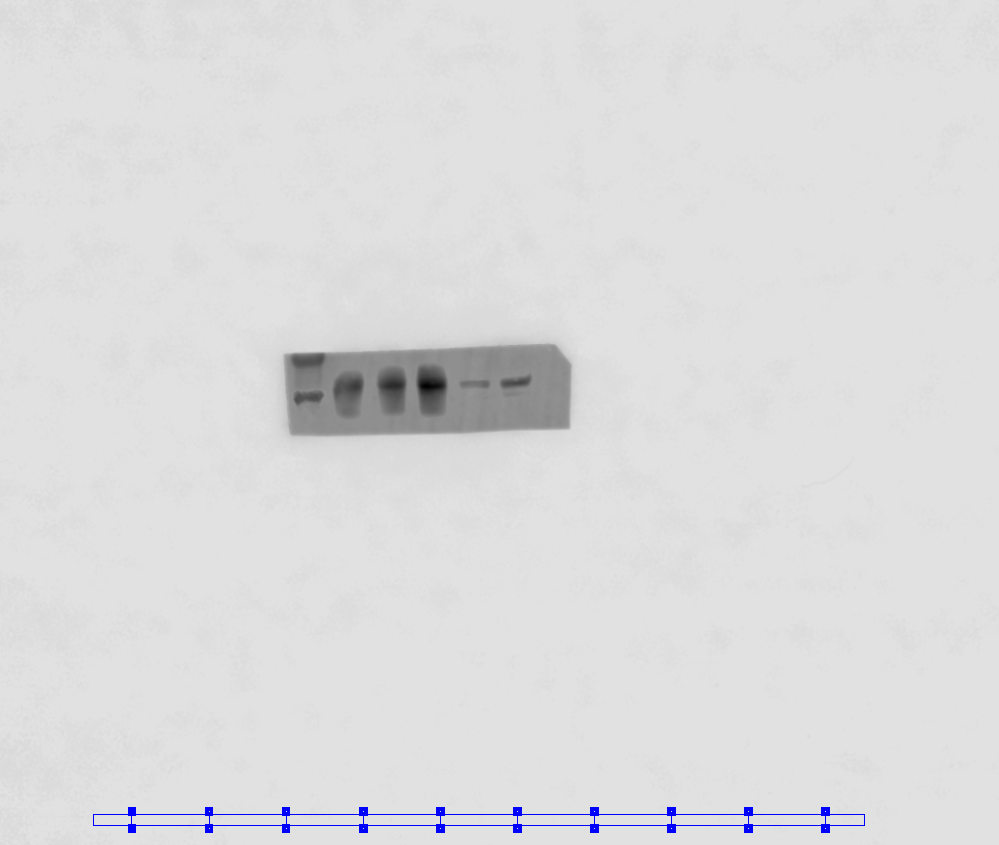

Supplement: Supplementary file 9 — Source data [file 41467_2023_43526_MOESM9_ESM.zip › Source Data/WB and Co-IP replications and quantification/Figuer.6e/replication_1/TADA3L.png]

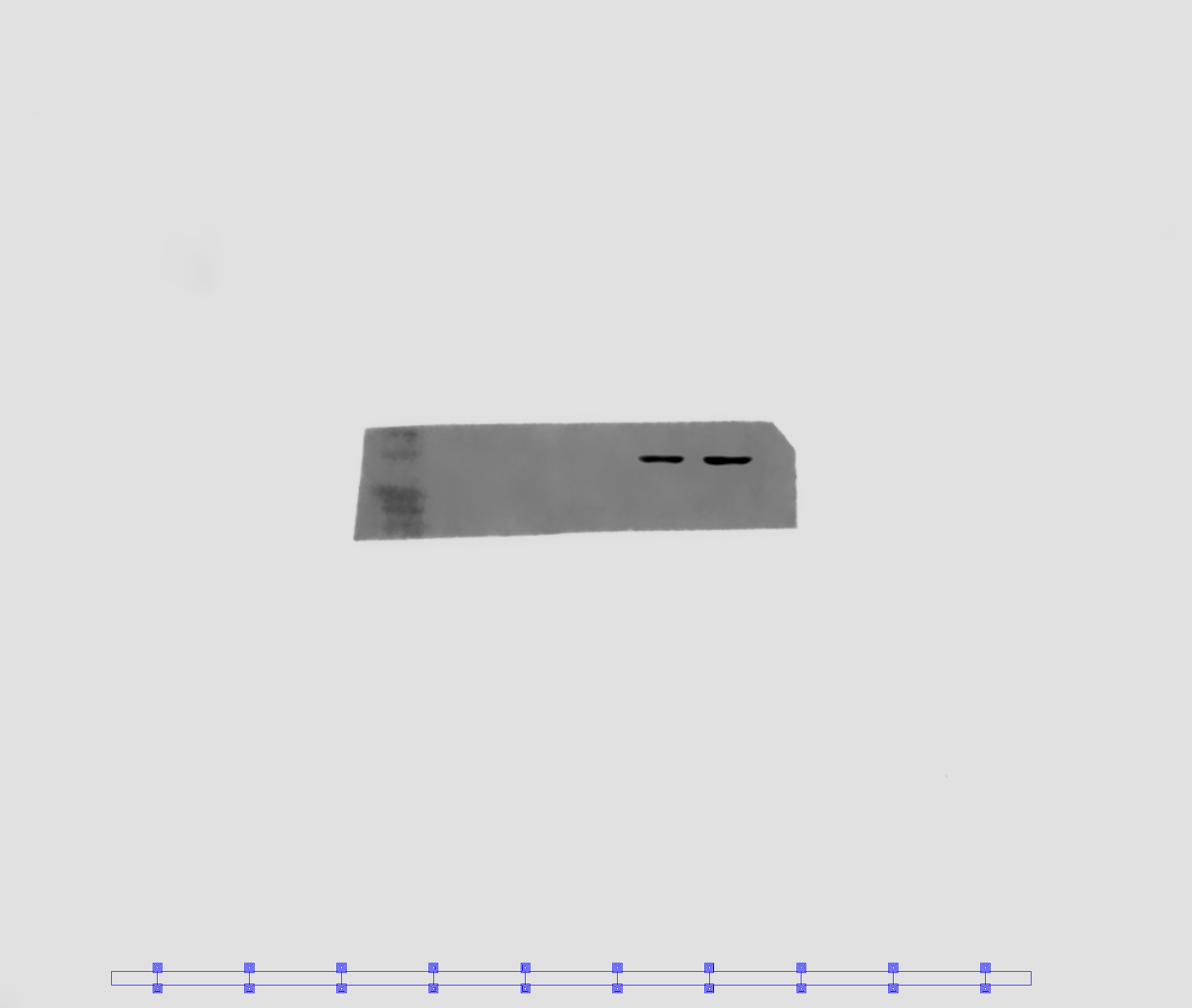

Supplement: Supplementary file 9 — Source data [file 41467_2023_43526_MOESM9_ESM.zip › Source Data/WB and Co-IP replications and quantification/Figuer.6e/replication_2/GAPDH.tif]

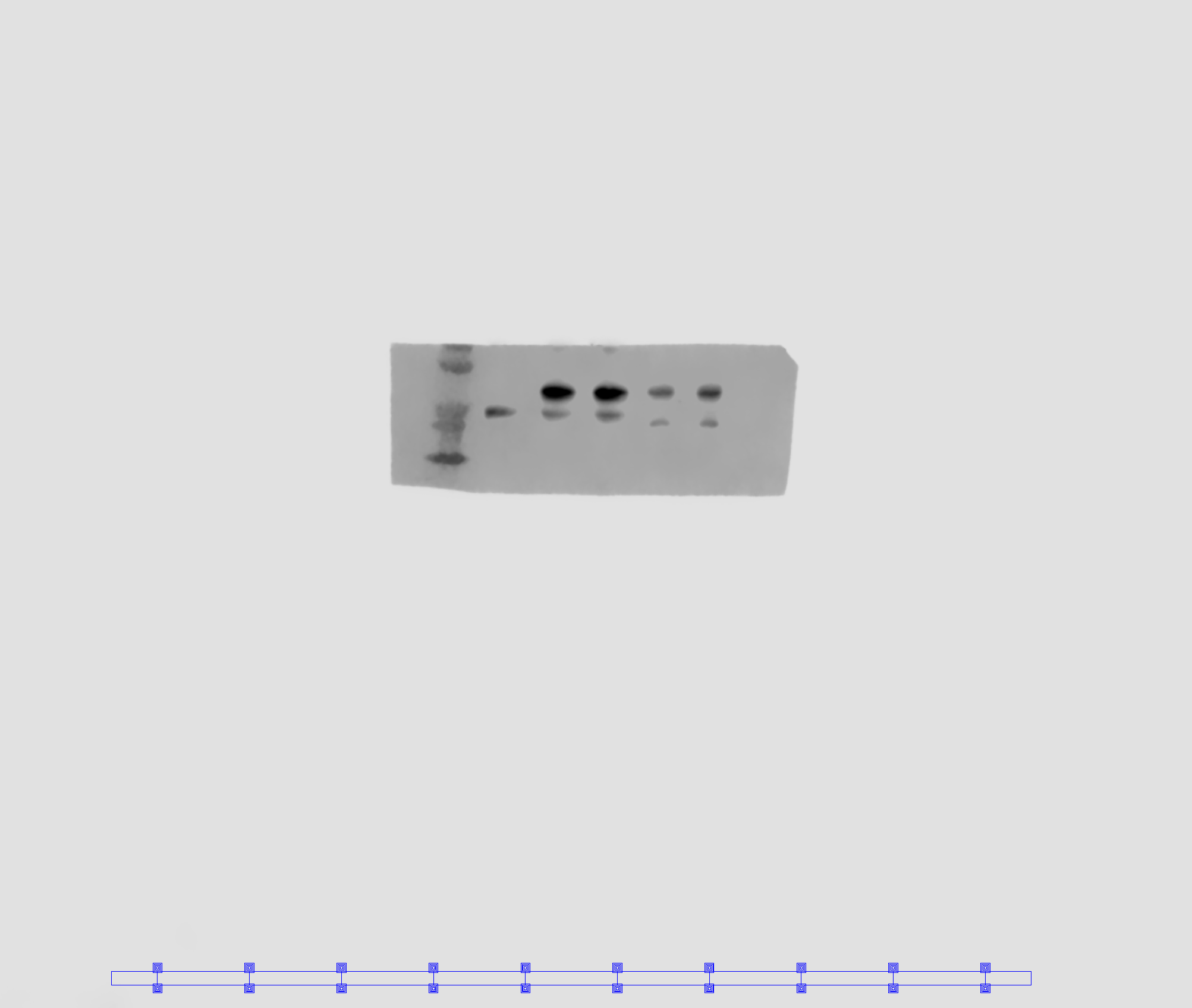

Supplement: Supplementary file 9 — Source data [file 41467_2023_43526_MOESM9_ESM.zip › Source Data/WB and Co-IP replications and quantification/Figuer.6e/replication_2/IP-TAP9B.tif]

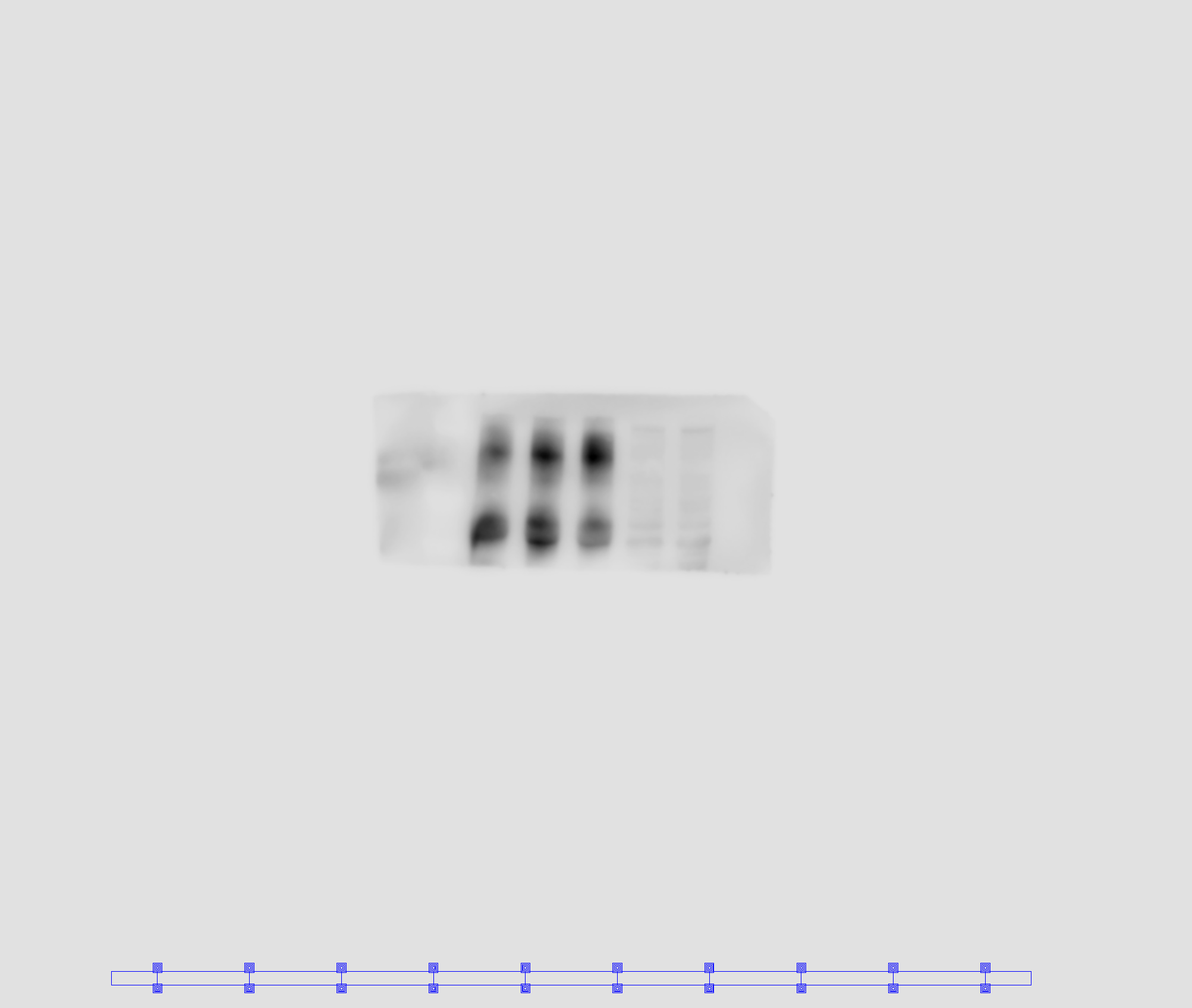

Supplement: Supplementary file 9 — Source data [file 41467_2023_43526_MOESM9_ESM.zip › Source Data/WB and Co-IP replications and quantification/Figuer.6e/replication_2/TADA3L.tif]

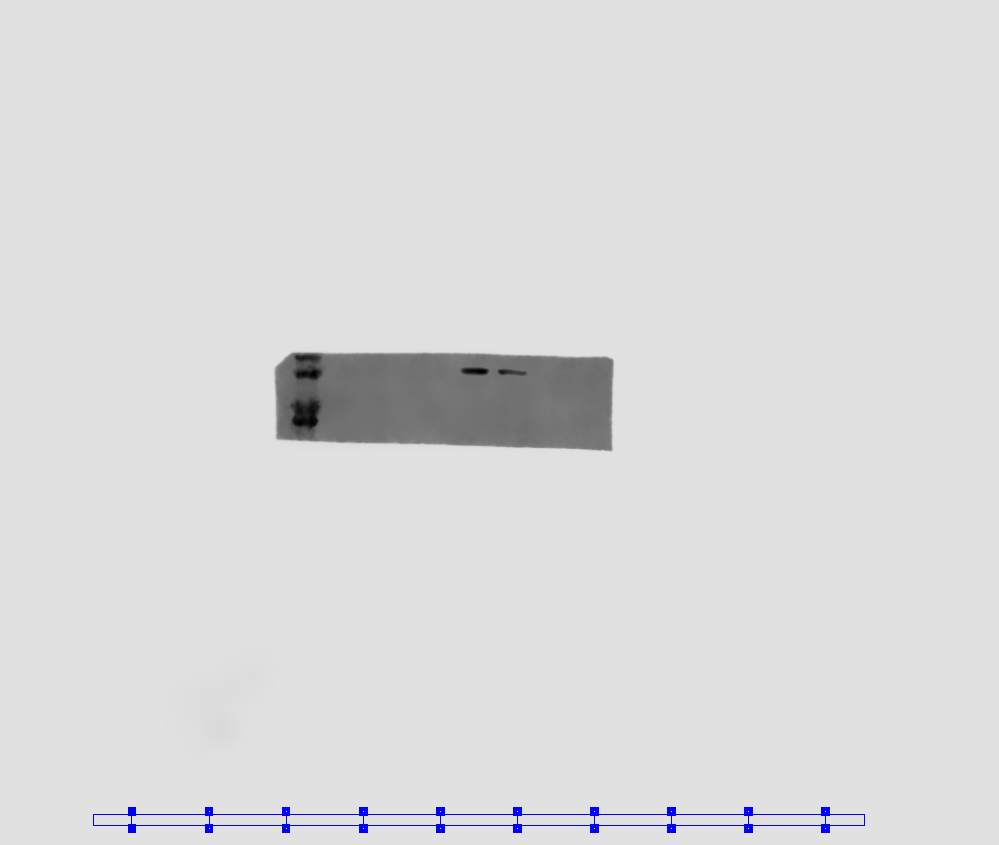

Supplement: Supplementary file 9 — Source data [file 41467_2023_43526_MOESM9_ESM.zip › Source Data/WB and Co-IP replications and quantification/Figuer.6e/replication_3/GAPDH.png]

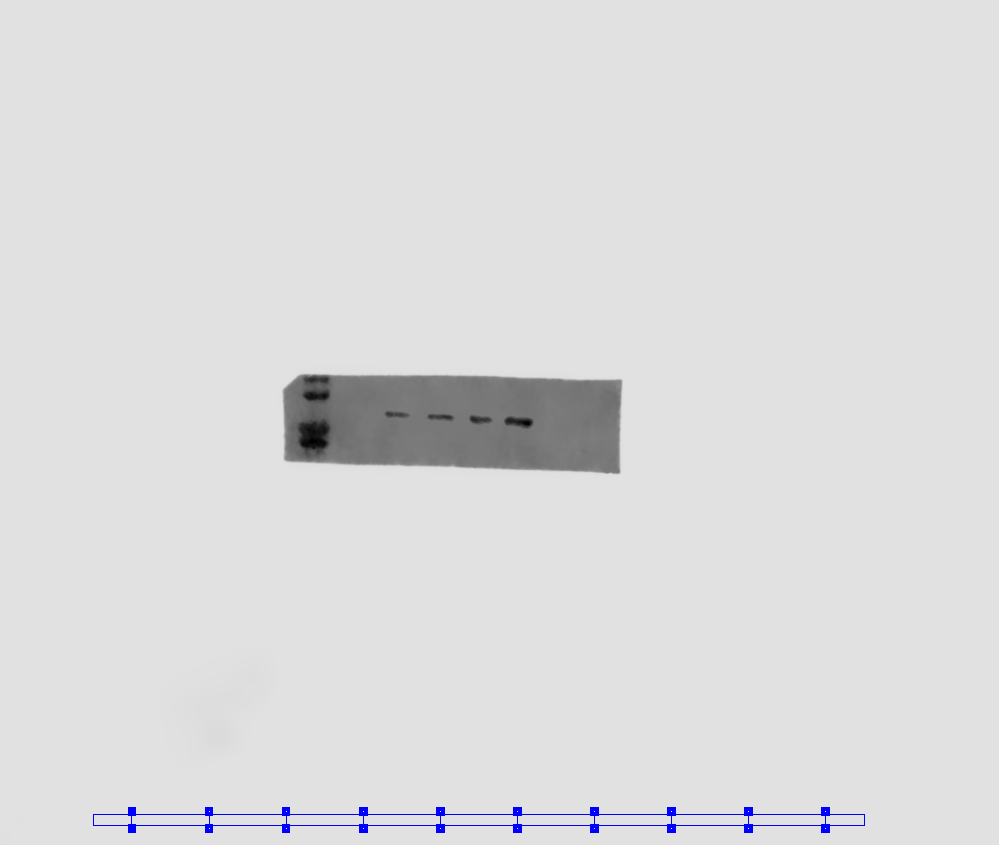

Supplement: Supplementary file 9 — Source data [file 41467_2023_43526_MOESM9_ESM.zip › Source Data/WB and Co-IP replications and quantification/Figuer.6e/replication_3/IP-TAF9B.png]

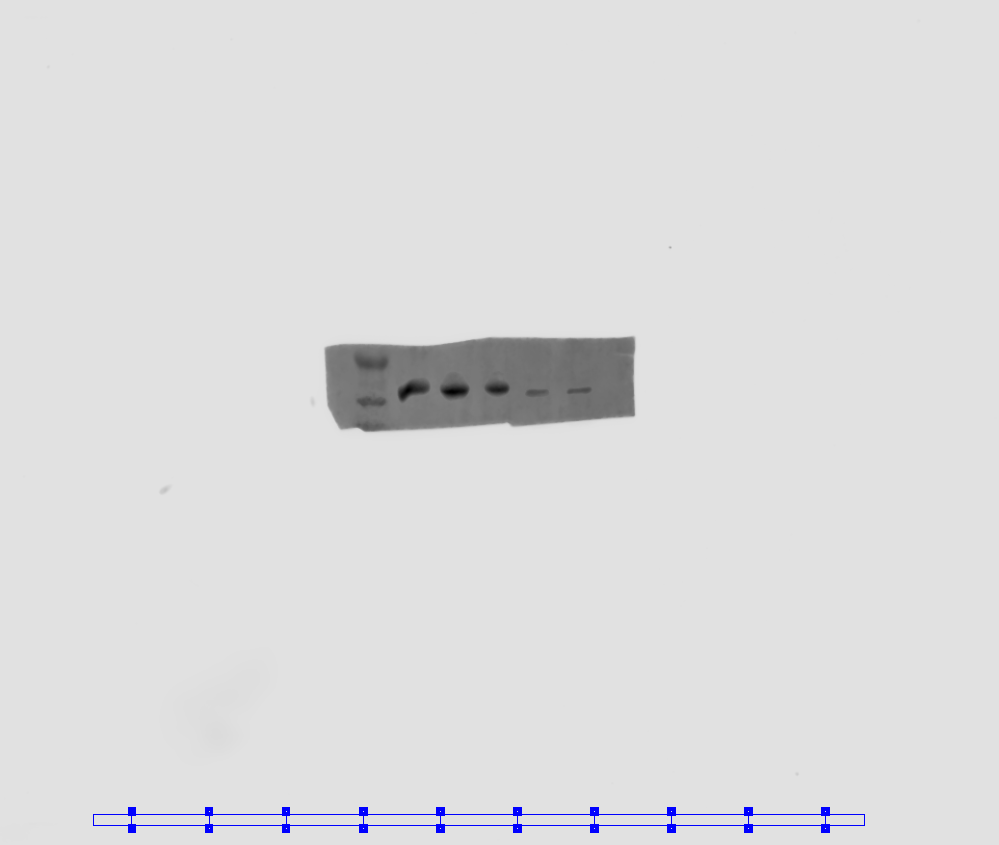

Supplement: Supplementary file 9 — Source data [file 41467_2023_43526_MOESM9_ESM.zip › Source Data/WB and Co-IP replications and quantification/Figuer.6e/replication_3/TADA3L.png]
